# Supplementary material for: CM-Dil Staining and SEC of Plasma as an Approach to Increase Sensitivity of Extracellular Nanovesicles Quantification by Bead-Assisted Flow Cytometry
Source: Membranes (Basel). 2021 Jul 13;11(7):526. doi: 10.3390/membranes11070526 (PMC8303825; doi:10.3390/membranes11070526)
Supplement: Supplementary file 1 [file membranes-11-00526-s001.zip › membranes-1255937-supplementary.pdf]

membranes

Supplementary information

CM-Dil Staining and SEC of Plasma as an Approach to Increase Sensitivity of Extracellular Nanovesicles Quantification by Bead-Assisted Flow Cytometry

Nadezhda Nikiforova [1], Maria Chumachenko [2], Inga Nazarova [1], Lidia Zabegina [1], Maria Slyusarenko [1,3], Elena Sidina [1] and Anastasia Malek [1,4,*]

1 Subcellular technology Laboratory., N.N. Petrov National Medical Center of Oncology, 197758 St. Petersburg, Russia; niki2naden_ka@mail.ru (N.N.); oblaka12@mail.ru (I.N.); lidusikza@yandex.ru (L.Z.); slusarenko_masha@mail.ru (M.S.); sidina@mail.ru (E.S.)
2 Deptment of Biochemistry, Belarusian State University, 220030 Minsk, Belarus; chumachenkomaria19@gmail.com (M.C.)
3 The Faculty of Physics, Saint-Petersburg State University, 199034 St. Petersburg, Russia;
4 Oncosystem Ltd., 121205 Moscow, Russia
* Correspondence: anastasia@malek.com; Tel.: +7-960-250-46-80

Figure S1: CM-Dil, SEC fraction: 5-6

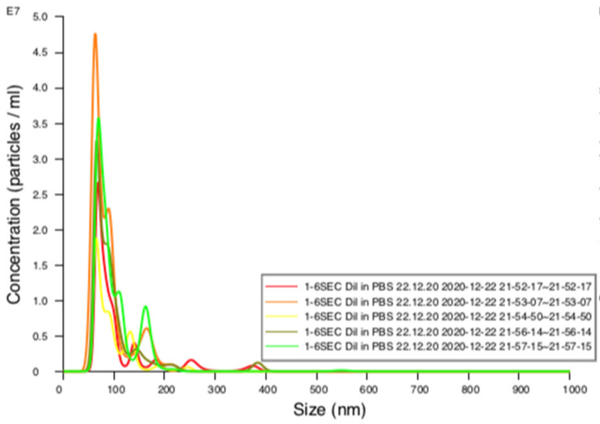

FTLA Concentration / Size graph for Experiment:
1-6SEC Dil in PBS 22.12.20 2020-12-22 21-51-35

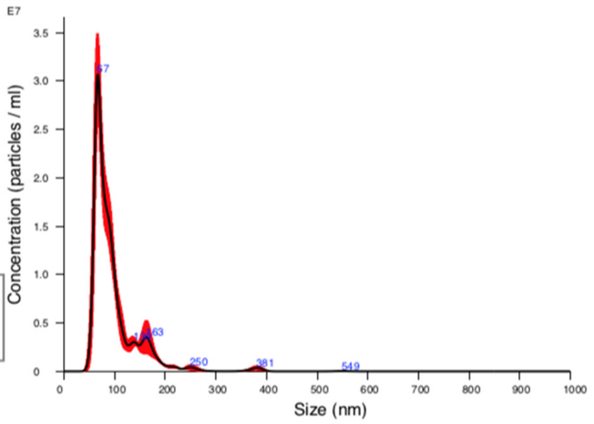

Averaged FTLA Concentration / Size for Experiment:
1-6SEC Dil in PBS 22.12.20 2020-12-22 21-51-35
Error bars indicate + / -1 standard error of the mean

Included Files

1-6SEC Dil in PBS 22.12.20 2020-12-22 21-52-17
1-6SEC Dil in PBS 22.12.20 2020-12-22 21-53-07
1-6SEC Dil in PBS 22.12.20 2020-12-22 21-54-50
1-6SEC Dil in PBS 22.12.20 2020-12-22 21-56-14
1-6SEC Dil in PBS 22.12.20 2020-12-22 21-57-15

Details

NTA Version:	NTA 3.4 Build 3.4.003
Script Used:	SOP Standard Measurement 09-51-35PM 22~
Time Captured:	21:51:35 22/12/2020
Operator:	Nadya
Pre-treatment:	
Sample Name:	1-6SEC Dil in PBS 22.12.20
Diluent:	PBS
Remarks:	

Capture Settings

Camera Type:	sCMOS
Laser Type:	Blue488
Camera Level:	15
Slider Shutter:	1206
Slider Gain:	245
FPS	25.0
Number of Frames:	749
Temperature:	25.3 - 25.5 ºC
Viscosity:	(Water) 0.878 - 0.883 cP
Dilution factor:	Dilution not recorded

Analysis Settings

Detect Threshold:	7
Blur Size:	Auto
Max Jump Distance:	Auto: 15.7 - 17.2 pix

Results

Stats: Merged Data

Mean:	96.7 nm
Mode:	66.6 nm
SD:	49.2 nm
D10:	61.0 nm
D50:	81.2 nm
D90:	158.2 nm

Stats: Mean +/- Standard Error

Mean:	97.6 +/- 3.2 nm
Mode:	66.4 +/- 1.2 nm
SD:	48.7 +/- 5.7 nm
D10:	61.8 +/- 1.5 nm
D50:	81.3 +/- 1.5 nm
D90:	159.2 +/- 8.5 nm
Concentration (Upgrade):	1.25e+09 +/- 1.95e+08 particles/ml
	73.9 +/- 10.1 particles/frame
	76.5 +/- 8.5 centres/frame

Figure S2: CM-Dil, SEC fraction: 7-8

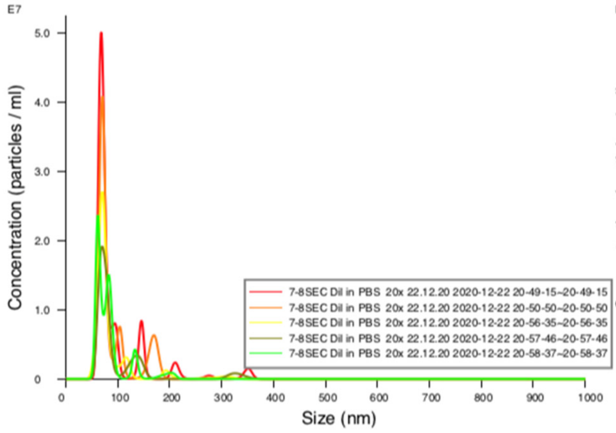

FTLA Concentration / Size graph for Experiment:
7-8SEC Dil in PBS 20x 22.12.20 2020-12-22 20-49-02

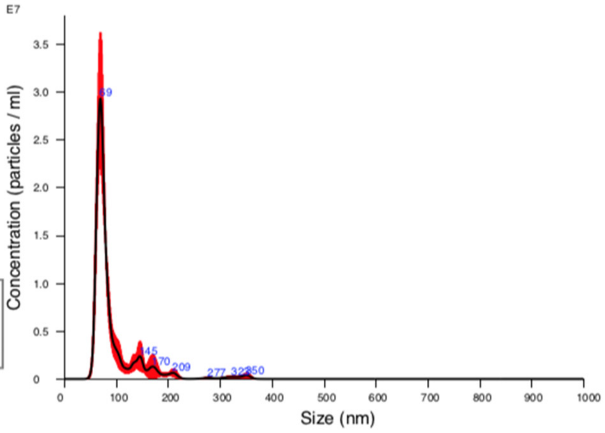

Averaged FTLA Concentration / Size for Experiment:
7-8SEC Dil in PBS 20x 22.12.20 2020-12-22 20-49-02
Error bars indicate + / -1 standard error of the mean

Included Files

7-8SEC Dil in PBS 20x 22.12.20 2020-12-22 20-49-15
7-8SEC Dil in PBS 20x 22.12.20 2020-12-22 20-50-50
7-8SEC Dil in PBS 20x 22.12.20 2020-12-22 20-56-35
7-8SEC Dil in PBS 20x 22.12.20 2020-12-22 20-57-46
7-8SEC Dil in PBS 20x 22.12.20 2020-12-22 20-58-37

Details

NTA Version:	NTA 3.4 Build 3.4.003
Script Used:	SOP Standard Measurement 08-49-02PM 22~
Time Captured:	20:49:02 22/12/2020
Operator:	Nadya
Pre-treatment:	
Sample Name:	7-8SEC Dil in PBS 20x 22.12.20
Diluent:	PBS
Remarks:	

Capture Settings

Camera Type:	sCMOS
Laser Type:	Blue488
Camera Level:	15
Slider Shutter:	1206
Slider Gain:	245
FPS	25.0
Number of Frames:	749
Temperature:	24.9 - 25.3 ºC
Viscosity:	(Water) 0.883 - 0.890 cP
Dilution factor:	Dilution not recorded

Analysis Settings

Detect Threshold:	17
Blur Size:	Auto
Max Jump Distance:	Auto: 16.2 - 18.1 pix

Results

Stats: Merged Data

Mean:	91.2 nm
Mode:	68.9 nm
SD:	49.1 nm
D10:	61.4 nm
D50:	73.6 nm
D90:	147.6 nm

Stats: Mean +/- Standard Error

Mean:	90.8 +/- 2.8 nm
Mode:	67.9 +/- 1.7 nm
SD:	47.0 +/- 5.6 nm
D10:	61.4 +/- 0.9 nm
D50:	73.9 +/- 0.7 nm
D90:	141.9 +/- 8.0 nm
Concentration (Upgrade):	7.95e+08 +/- 1.02e+08 particles/ml
	27.2 +/- 3.5 particles/frame
	28.6 +/- 3.6 centres/frame

Figure S3: CM-DiI, SEC fraction: 9-11

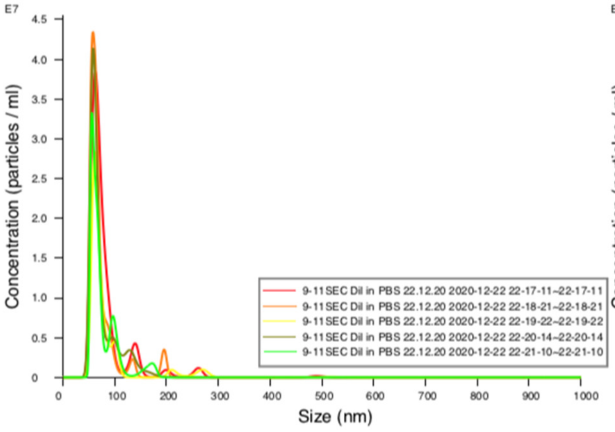

FTLA Concentration / Size graph for Experiment:
9-11SEC Dil in PBS 22.12.20 2020-12-22 22-16-51

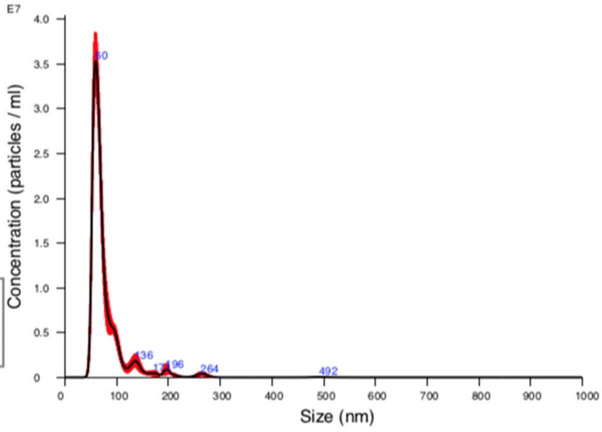

Averaged FTLA Concentration / Size for Experiment:
9-11SEC Dil in PBS 22.12.20 2020-12-22 22-16-51
Error bars indicate + / -1 standard error of the mean

Included Files

9-11SEC Dil in PBS 22.12.20 2020-12-22 22-17-11
9-11SEC Dil in PBS 22.12.20 2020-12-22 22-18-21
9-11SEC Dil in PBS 22.12.20 2020-12-22 22-19-22
9-11SEC Dil in PBS 22.12.20 2020-12-22 22-20-14
9-11SEC Dil in PBS 22.12.20 2020-12-22 22-21-10

Details

NTA Version:	NTA 3.4 Build 3.4.003
Script Used:	SOP Standard Measurement 10-16-51PM 22~
Time Captured:	22:16:51 22/12/2020
Operator:	Nadya
Pre-treatment:	
Sample Name:	9-11SEC Dil in PBS 22.12.20
Diluent:	PBS
Remarks:	

Capture Settings

Camera Type:	sCMOS
Laser Type:	Blue488
Camera Level:	15
Slider Shutter:	1206
Slider Gain:	245
FPS	25.0
Number of Frames:	749
Temperature:	25.7 - 25.9 ºC
Viscosity:	(Water) 0.871 - 0.874 cP
Dilution factor:	Dilution not recorded

Analysis Settings

Detect Threshold:	7
Blur Size:	Auto
Max Jump Distance:	Auto: 17.2 - 18.5 pix

Results

Stats: Merged Data

Mean:	78.2 nm
Mode:	59.2 nm
SD:	40.5 nm
D10:	53.7 nm
D50:	65.6 nm
D90:	112.4 nm

Stats: Mean +/- Standard Error

Mean:	78.3 +/- 2.3 nm
Mode:	59.3 +/- 1.2 nm
SD:	38.9 +/- 6.4 nm
D10:	53.9 +/- 0.5 nm
D50:	65.6 +/- 1.0 nm
D90:	112.6 +/- 6.0 nm
Concentration (Upgrade):	9.78e+08 +/- 9.32e+07 particles/ml
	52.3 +/- 5.3 particles/frame
	52.7 +/- 4.7 centres/frame

Figure S4: CM-Dil, SEC fraction: 12-13

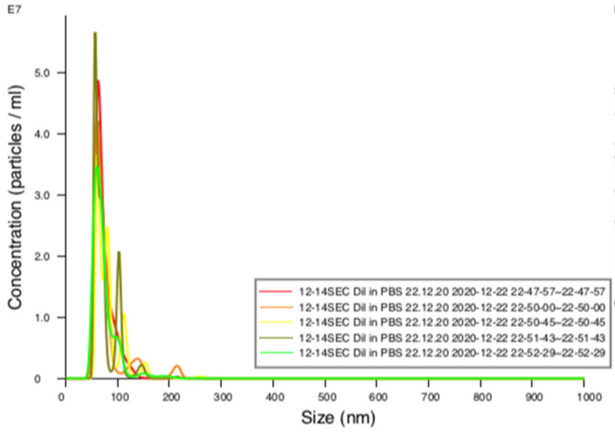

FTLA Concentration / Size graph for Experiment:
12-14SEC Dil in PBS 22.12.20 2020-12-22 22-46-45

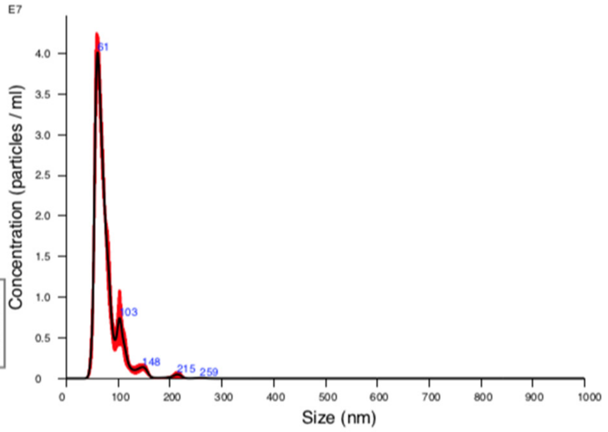

Averaged FTLA Concentration / Size for Experiment:
12-14SEC Dil in PBS 22.12.20 2020-12-22 22-46-45
Error bars indicate + / -1 standard error of the mean

Included Files

12-14SEC Dil in PBS 22.12.20 2020-12-22 22-47-57
12-14SEC Dil in PBS 22.12.20 2020-12-22 22-50-00
12-14SEC Dil in PBS 22.12.20 2020-12-22 22-50-45
12-14SEC Dil in PBS 22.12.20 2020-12-22 22-51-43
12-14SEC Dil in PBS 22.12.20 2020-12-22 22-52-29

Details

NTA Version:	NTA 3.4 Build 3.4.003
Script Used:	SOP Standard Measurement 10-46-45PM 22~
Time Captured:	22:46:45 22/12/2020
Operator:	Nadya
Pre-treatment:	
Sample Name:	12-14SEC Dil in PBS 22.12.20
Diluent:	PBS
Remarks:	

Capture Settings

Camera Type:	sCMOS
Laser Type:	Blue488
Camera Level:	15
Slider Shutter:	1206
Slider Gain:	245
FPS	25.0
Number of Frames:	749
Temperature:	26.1 - 26.2 ºC
Viscosity:	(Water) 0.866 - 0.868 cP
Dilution factor:	Dilution not recorded

Analysis Settings

Detect Threshold:	7
Blur Size:	Auto
Max Jump Distance:	Auto: 17.7 - 18.3 pix

Results

Stats: Merged Data

Mean:	76.0 nm
Mode:	60.2 nm
SD:	26.3 nm
D10:	55.4 nm
D50:	67.9 nm
D90:	106.5 nm

Stats: Mean +/- Standard Error

Mean:	76.2 +/- 1.7 nm
Mode:	60.3 +/- 1.0 nm
SD:	25.7 +/- 2.5 nm
D10:	55.6 +/- 1.0 nm
D50:	68.6 +/- 1.7 nm
D90:	109.2 +/- 5.3 nm
Concentration (Upgrade):	1.13e+09 +/- 4.53e+07 particles/ml
	50.0 +/- 1.7 particles/frame
	51.0 +/- 1.8 centres/frame

Figure S5: CM-Dil, SEC fraction: 16-21

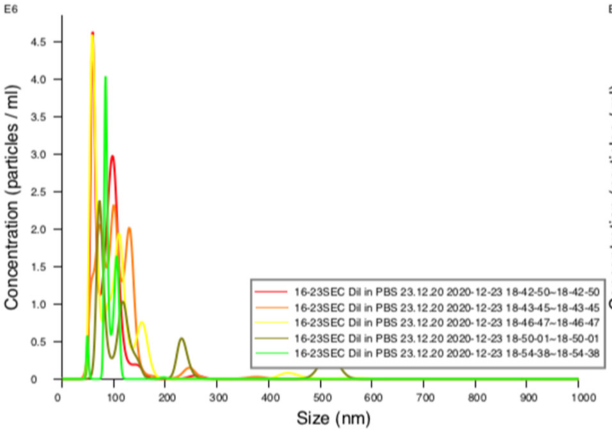

FTLA Concentration / Size graph for Experiment:
16-23SEC Dil in PBS 23.12.20 2020-12-23 18-41-47

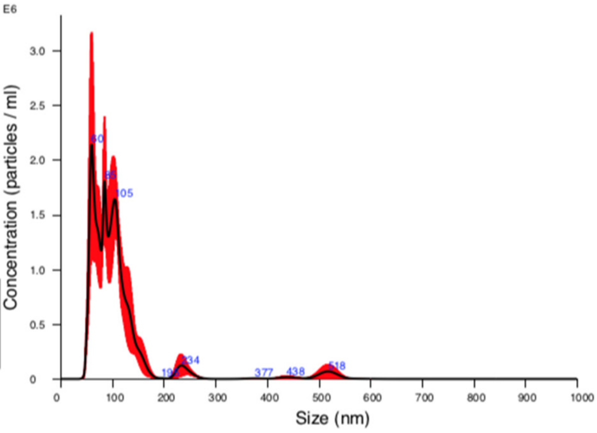

Averaged FTLA Concentration / Size for Experiment:
16-23SEC Dil in PBS 23.12.20 2020-12-23 18-41-47
Error bars indicate + / -1 standard error of the mean

Included Files

16-23SEC Dil in PBS 23.12.20 2020-12-23 18-42-50
16-23SEC Dil in PBS 23.12.20 2020-12-23 18-43-45
16-23SEC Dil in PBS 23.12.20 2020-12-23 18-46-47
16-23SEC Dil in PBS 23.12.20 2020-12-23 18-50-01
16-23SEC Dil in PBS 23.12.20 2020-12-23 18-54-38

Details

NTA Version:	NTA 3.4 Build 3.4.003
Script Used:	SOP Standard Measurement 06-41-47PM 23~
Time Captured:	18:41:47 23/12/2020
Operator:	Nadya
Pre-treatment:	
Sample Name:	16-23SEC Dil in PBS 23.12.20
Diluent:	PBS
Remarks:	

Capture Settings

Camera Type:	sCMOS
Laser Type:	Blue488
Camera Level:	16
Slider Shutter:	1300
Slider Gain:	295
FPS	25.0
Number of Frames:	749
Temperature:	24.5 - 25.0 ºC
Viscosity:	(Water) 0.888 - 0.900 cP
Dilution factor:	Dilution not recorded

Analysis Settings

Detect Threshold:	7
Blur Size:	Auto
Max Jump Distance:	Auto: 14.2 - 19.4 pix

Results

Stats: Merged Data

Mean:	110.4 nm
Mode:	59.3 nm
SD:	80.9 nm
D10:	58.7 nm
D50:	93.1 nm
D90:	148.3 nm

Stats: Mean +/- Standard Error

Mean:	112.7 +/- 16.3 nm
Mode:	75.1 +/- 7.9 nm
SD:	62.6 +/- 24.0 nm
D10:	65.4 +/- 4.6 nm
D50:	95.7 +/- 5.2 nm
D90:	204.5 +/- 76.5 nm
Concentration (Upgrade):	1.30e+08 +/- 2.18e+07 particles/ml
	9.2 +/- 1.5 particles/frame
	9.6 +/- 1.5 centres/frame

Figure S6: PL, SEC fraction: 5-6

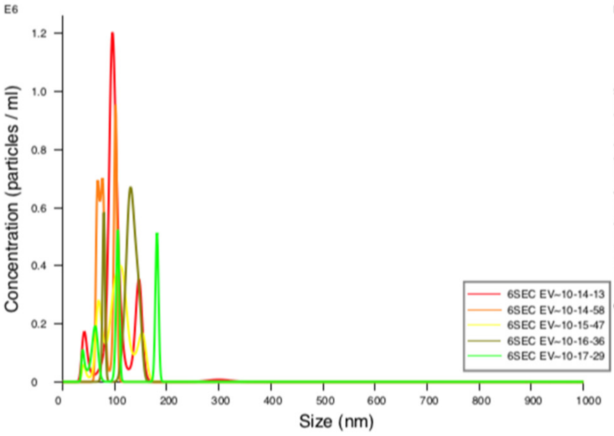

FTLA Concentration / Size graph for Experiment:
6SEC EVs 20x 25.11.20 2020-11-25 10-13-30

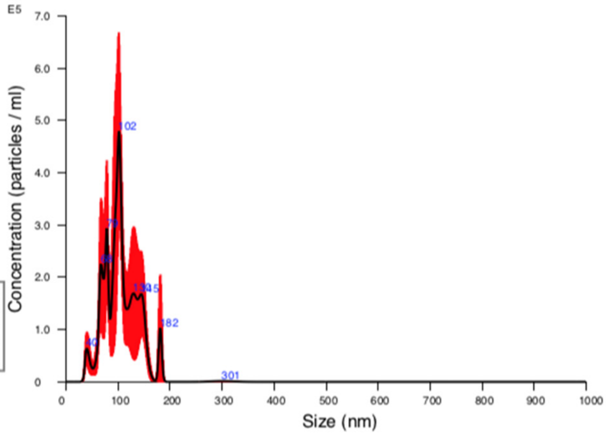

Averaged FTLA Concentration / Size for Experiment:
6SEC EVs 20x 25.11.20 2020-11-25 10-13-30
Error bars indicate + / -1 standard error of the mean

Included Files

6SEC EVs 20x 25.11.20 2020-11-25 10-14-13
6SEC EVs 20x 25.11.20 2020-11-25 10-14-58
6SEC EVs 20x 25.11.20 2020-11-25 10-15-47
6SEC EVs 20x 25.11.20 2020-11-25 10-16-36
6SEC EVs 20x 25.11.20 2020-11-25 10-17-29

Details

NTA Version:	NTA 3.4 Build 3.4.003
Script Used:	SOP Standard Measurement 10-13-30AM 25~
Time Captured:	10:13:30 25/11/2020
Operator:	Nadya24.11.20
Pre-treatment:	
Sample Name:	6SEC EVs 20x 25.11.20
Diluent:	PBS
Remarks:	

Capture Settings

Camera Type:	sCMOS
Laser Type:	Blue488
Camera Level:	16
Slider Shutter:	1300
Slider Gain:	295
FPS	25.0
Number of Frames:	749
Temperature:	22.9 ºC
Viscosity:	(Water) 0.931 - 0.933 cP
Dilution factor:	Dilution not recorded

Analysis Settings

Detect Threshold:	5
Blur Size:	Auto
Max Jump Distance:	Auto: 15.1 pix

Results

Stats: Merged Data

Mean:	105.8 nm
Mode:	101.8 nm
SD:	33.4 nm
D10:	67.4 nm
D50:	102.0 nm
D90:	147.4 nm

Stats: Mean +/- Standard Error

Mean:	106.4 +/- 6.8 nm
Mode:	108.9 +/- 5.9 nm
SD:	30.2 +/- 6.2 nm
D10:	69.1 +/- 4.9 nm
D50:	103.8 +/- 8.3 nm
D90:	145.3 +/- 12.6 nm
Concentration (Upgrade):	2.18e+07 +/- 3.43e+06 particles/ml
	1.7 +/- 0.2 particles/frame
	2.1 +/- 0.3 centres/frame

Concentration measurements may be unreliable
See summary file for more info

Figure S7: PL, SEC fraction: 7

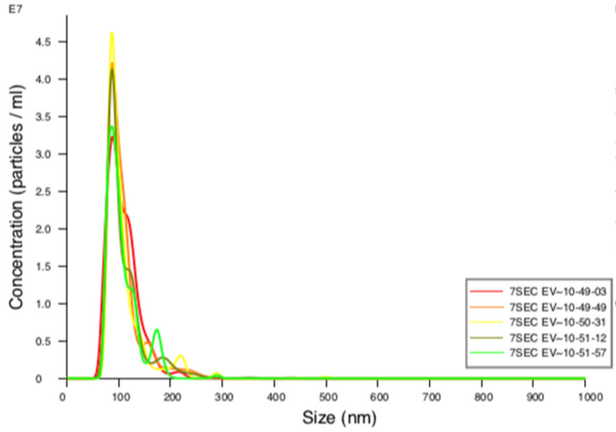

FTLA Concentration / Size graph for Experiment:
7SEC EVs 20x 25.11.20 2020-11-25 10-48-28

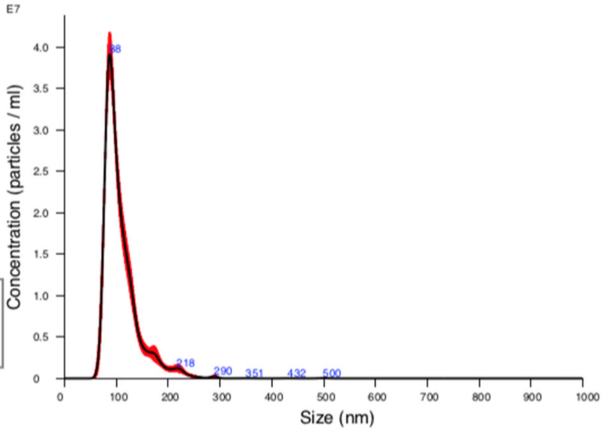

Averaged FTLA Concentration / Size for Experiment:
7SEC EVs 20x 25.11.20 2020-11-25 10-48-28
Error bars indicate + / -1 standard error of the mean

Included Files

7SEC EVs 20x 25.11.20 2020-11-25 10-49-03
7SEC EVs 20x 25.11.20 2020-11-25 10-49-49
7SEC EVs 20x 25.11.20 2020-11-25 10-50-31
7SEC EVs 20x 25.11.20 2020-11-25 10-51-12
7SEC EVs 20x 25.11.20 2020-11-25 10-51-57

Details

NTA Version:	NTA 3.4 Build 3.4.003
Script Used:	SOP Standard Measurement 10-48-28AM 25~
Time Captured:	10:48:28 25/11/2020
Operator:	Nadya24.11.20
Pre-treatment:	
Sample Name:	7SEC EVs 20x 25.11.20
Diluent:	PBS
Remarks:	

Capture Settings

Camera Type:	sCMOS
Laser Type:	Blue488
Camera Level:	13
Slider Shutter:	1232
Slider Gain:	175
FPS	25.0
Number of Frames:	749
Temperature:	24.3 - 24.5 ºC
Viscosity:	(Water) 0.899 - 0.902 cP
Dilution factor:	Dilution not recorded

Analysis Settings

Detect Threshold:	17
Blur Size:	Auto
Max Jump Distance:	Auto: 14.8 - 15.1 pix

Results

Stats: Merged Data

Mean:	108.8 nm
Mode:	87.2 nm
SD:	35.7 nm
D10:	78.4 nm
D50:	98.5 nm
D90:	151.7 nm

Stats: Mean +/- Standard Error

Mean:	108.8 +/- 0.6 nm
Mode:	87.3 +/- 0.3 nm
SD:	35.4 +/- 2.3 nm
D10:	78.4 +/- 0.7 nm
D50:	98.6 +/- 0.9 nm
D90:	153.5 +/- 2.3 nm
Concentration (Upgrade):	1.75e+09 +/- 5.80e+07 particles/ml
	61.9 +/- 2.5 particles/frame
	63.0 +/- 2.5 centres/frame

Figure S8: PL, SEC fraction: 8

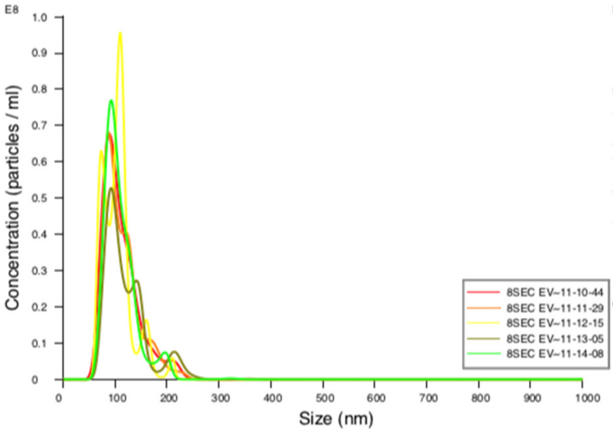

FTLA Concentration / Size graph for Experiment:
8SEC EVs 20x 25.11.20 2020-11-25 11-10-22

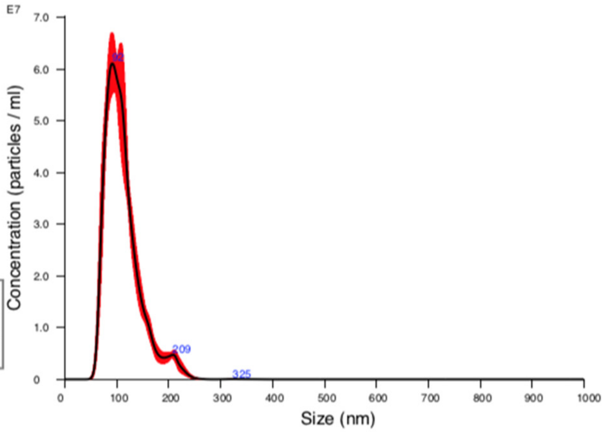

Averaged FTLA Concentration / Size for Experiment:
8SEC EVs 20x 25.11.20 2020-11-25 11-10-22
Error bars indicate + / -1 standard error of the mean

Included Files

8SEC EVs 20x 25.11.20 2020-11-25 11-10-44
8SEC EVs 20x 25.11.20 2020-11-25 11-11-29
8SEC EVs 20x 25.11.20 2020-11-25 11-12-15
8SEC EVs 20x 25.11.20 2020-11-25 11-13-05
8SEC EVs 20x 25.11.20 2020-11-25 11-14-08

Details

NTA Version:	NTA 3.4 Build 3.4.003
Script Used:	SOP Standard Measurement 11-10-22AM 25~
Time Captured:	11:10:22 25/11/2020
Operator:	Nadya24.11.20
Pre-treatment:	
Sample Name:	8SEC EVs 20x 25.11.20
Diluent:	PBS
Remarks:	

Capture Settings

Camera Type:	sCMOS
Laser Type:	Blue488
Camera Level:	13
Slider Shutter:	1232
Slider Gain:	175
FPS	25.0
Number of Frames:	749
Temperature:	24.4 - 24.4 ºC
Viscosity:	(Water) 0.900 - 0.901 cP
Dilution factor:	Dilution not recorded

Analysis Settings

Detect Threshold:	17
Blur Size:	Auto
Max Jump Distance:	Auto: 13.9 - 14.9 pix

Results

Stats: Merged Data

Mean:	111.2 nm
Mode:	91.3 nm
SD:	34.4 nm
D10:	75.8 nm
D50:	104.0 nm
D90:	156.5 nm

Stats: Mean +/- Standard Error

Mean:	111.6 +/- 2.4 nm
Mode:	94.2 +/- 4.0 nm
SD:	34.2 +/- 1.9 nm
D10:	76.4 +/- 1.6 nm
D50:	104.4 +/- 1.1 nm
D90:	157.4 +/- 5.1 nm
Concentration (Upgrade):	4.03e+09 +/- 1.97e+08 particles/ml
	190.3 +/- 6.3 particles/frame
	166.7 +/- 4.0 centres/frame

Figure S9: PL, SEC fraction: 9

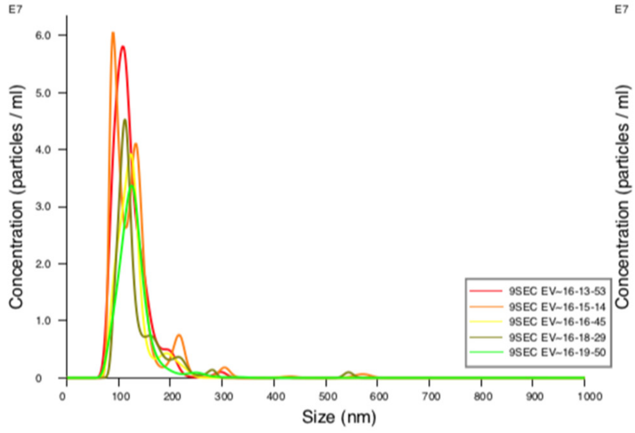

FTLA Concentration / Size graph for Experiment:
9SEC EVs 20x-1 24.11.20 2020-11-24 16-13-02

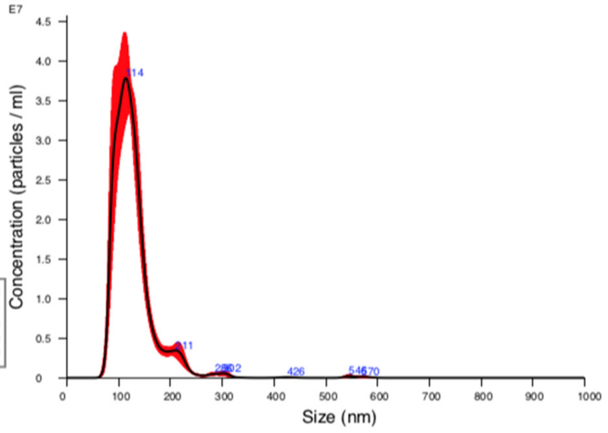

Averaged FTLA Concentration / Size for Experiment:
9SEC EVs 20x-1 24.11.20 2020-11-24 16-13-02
Error bars indicate + / -1 standard error of the mean

Included Files

9SEC EVs 20x-1 24.11.20 2020-11-24 16-13-53
9SEC EVs 20x-1 24.11.20 2020-11-24 16-15-14
9SEC EVs 20x-1 24.11.20 2020-11-24 16-16-45
9SEC EVs 20x-1 24.11.20 2020-11-24 16-18-29
9SEC EVs 20x-1 24.11.20 2020-11-24 16-19-50

Details

NTA Version:	NTA 3.4 Build 3.4.003
Script Used:	SOP Standard Measurement 04-13-02PM 24~
Time Captured:	16:13:02 24/11/2020
Operator:	Nadya24.11.20
Pre-treatment:	
Sample Name:	9SEC EVs 20x-1 24.11.20
Diluent:	PBS
Remarks:	

Capture Settings

Camera Type:	sCMOS
Laser Type:	Blue488
Camera Level:	12
Slider Shutter:	1200
Slider Gain:	125
FPS	25.0
Number of Frames:	1498
Temperature:	25.0 - 25.1 ºC
Viscosity:	(Water) 0.886 - 0.889 cP
Dilution factor:	Dilution not recorded

Analysis Settings

Detect Threshold:	17
Blur Size:	Auto
Max Jump Distance:	Auto: 13.7 - 14.2 pix

Results

Stats: Merged Data

Mean:	127.9 nm
Mode:	113.6 nm
SD:	46.6 nm
D10:	89.6 nm
D50:	118.8 nm
D90:	172.4 nm

Stats: Mean +/- Standard Error

Mean:	128.7 +/- 2.3 nm
Mode:	111.7 +/- 6.4 nm
SD:	44.3 +/- 6.0 nm
D10:	91.9 +/- 2.1 nm
D50:	119.3 +/- 1.9 nm
D90:	176.2 +/- 6.6 nm
Concentration (Upgrade):	2.53e+09 +/- 3.20e+08 particles/ml
	89.6 +/- 14.7 particles/frame
	89.7 +/- 14.1 centres/frame

Figure S10: PL, SEC fraction: 10

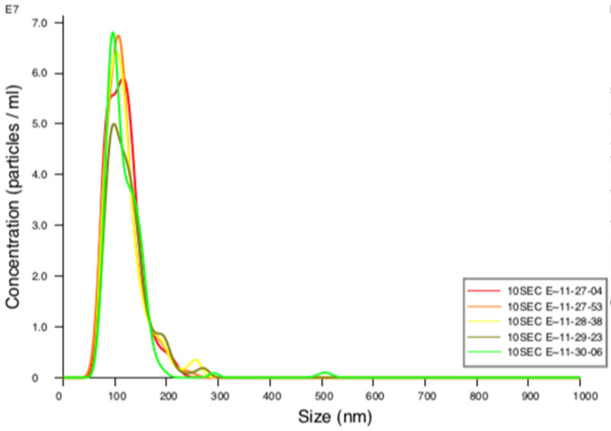

FTLA Concentration / Size graph for Experiment:
10SEC EVs 20x 25.11.20 2020-11-25 11-26-39

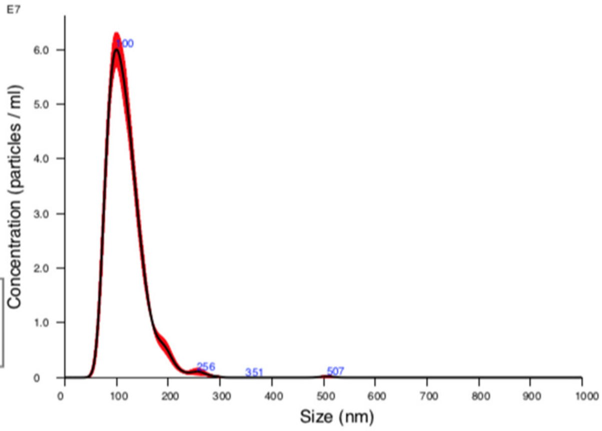

Averaged FTLA Concentration / Size for Experiment:
10SEC EVs 20x 25.11.20 2020-11-25 11-26-39
Error bars indicate + / -1 standard error of the mean

Included Files

10SEC EVs 20x 25.11.20 2020-11-25 11-27-04
10SEC EVs 20x 25.11.20 2020-11-25 11-27-53
10SEC EVs 20x 25.11.20 2020-11-25 11-28-38
10SEC EVs 20x 25.11.20 2020-11-25 11-29-23
10SEC EVs 20x 25.11.20 2020-11-25 11-30-06

Details

NTA Version:	NTA 3.4 Build 3.4.003
Script Used:	SOP Standard Measurement 11-26-39AM 25~
Time Captured:	11:26:39 25/11/2020
Operator:	Nadya24.11.20
Pre-treatment:	
Sample Name:	10SEC EVs 20x 25.11.20
Diluent:	PBS
Remarks:	

Capture Settings

Camera Type:	sCMOS
Laser Type:	Blue488
Camera Level:	12
Slider Shutter:	1200
Slider Gain:	125
FPS	25.0
Number of Frames:	749
Temperature:	24.3 - 24.3 ºC
Viscosity:	(Water) 0.902 - 0.904 cP
Dilution factor:	Dilution not recorded

Analysis Settings

Detect Threshold:	17
Blur Size:	Auto
Max Jump Distance:	Auto: 14.1 - 14.8 pix

Results

Stats: Merged Data

Mean:	117.9 nm
Mode:	99.7 nm
SD:	38.1 nm
D10:	79.8 nm
D50:	111.4 nm
D90:	161.6 nm

Stats: Mean +/- Standard Error

Mean:	118.1 +/- 1.4 nm
Mode:	103.9 +/- 3.5 nm
SD:	37.9 +/- 2.3 nm
D10:	79.9 +/- 0.9 nm
D50:	111.6 +/- 1.2 nm
D90:	163.2 +/- 3.7 nm
Concentration (Upgrade):	4.37e+09 +/- 1.42e+08 particles/ml
	177.9 +/- 4.9 particles/frame
	173.1 +/- 3.9 centres/frame

Figure S11: PL, SEC fraction: 11

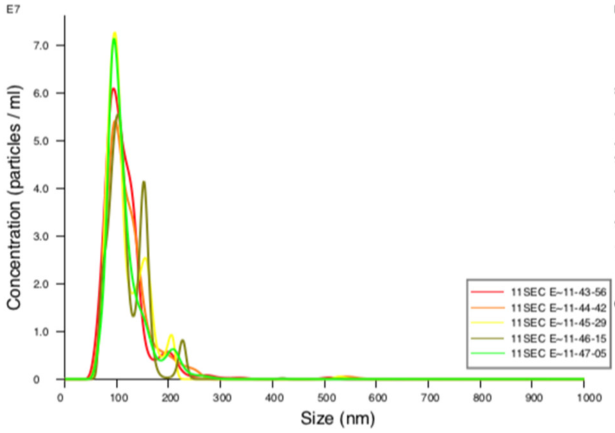

FTLA Concentration / Size graph for Experiment:
11SEC EVs 20x 25.11.20 2020-11-25 11-43-10

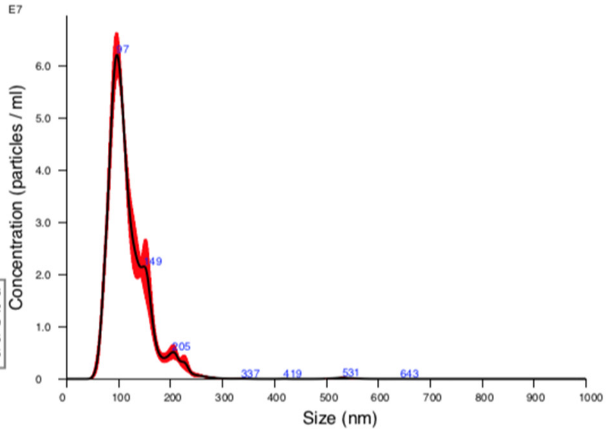

Averaged FTLA Concentration / Size for Experiment:
11SEC EVs 20x 25.11.20 2020-11-25 11-43-10
Error bars indicate + / -1 standard error of the mean

Included Files

11SEC EVs 20x 25.11.20 2020-11-25 11-43-56
11SEC EVs 20x 25.11.20 2020-11-25 11-44-42
11SEC EVs 20x 25.11.20 2020-11-25 11-45-29
11SEC EVs 20x 25.11.20 2020-11-25 11-46-15
11SEC EVs 20x 25.11.20 2020-11-25 11-47-05

Details

NTA Version:	NTA 3.4 Build 3.4.003
Script Used:	SOP Standard Measurement 11-43-10AM 25~
Time Captured:	11:43:10 25/11/2020
Operator:	Nadya24.11.20
Pre-treatment:	
Sample Name:	11SEC EVs 20x 25.11.20
Diluent:	PBS
Remarks:	

Capture Settings

Camera Type:	sCMOS
Laser Type:	Blue488
Camera Level:	12
Slider Shutter:	1200
Slider Gain:	125
FPS	25.0
Number of Frames:	749
Temperature:	24.3 - 24.4 ºC
Viscosity:	(Water) 0.901 - 0.904 cP
Dilution factor:	Dilution not recorded

Analysis Settings

Detect Threshold:	17
Blur Size:	Auto
Max Jump Distance:	Auto: 14.2 - 14.7 pix

Results

Stats: Merged Data

Mean:	118.1 nm
Mode:	96.6 nm
SD:	43.4 nm
D10:	79.6 nm
D50:	107.4 nm
D90:	164.3 nm

Stats: Mean +/- Standard Error

Mean:	118.1 +/- 1.3 nm
Mode:	97.0 +/- 1.5 nm
SD:	42.9 +/- 2.7 nm
D10:	79.7 +/- 0.8 nm
D50:	107.6 +/- 1.4 nm
D90:	164.5 +/- 3.1 nm
Concentration (Upgrade):	3.79e+09 +/- 1.04e+08 particles/ml
	148.8 +/- 6.4 particles/frame
	152.7 +/- 6.1 centres/frame

Figure S12: PL, SEC fraction: 12

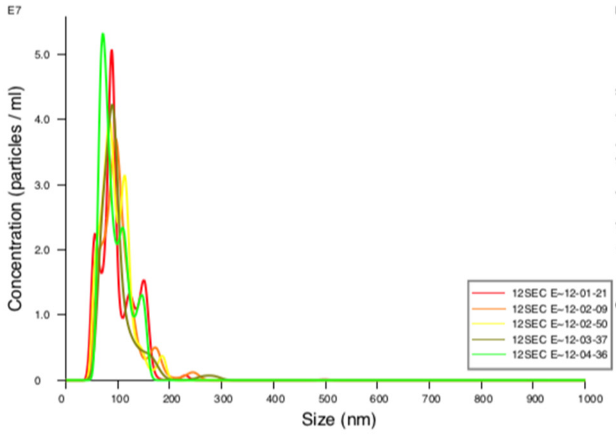

FTLA Concentration / Size graph for Experiment:
12SEC EVs 20x 25.11.20 2020-11-25 12-00-58

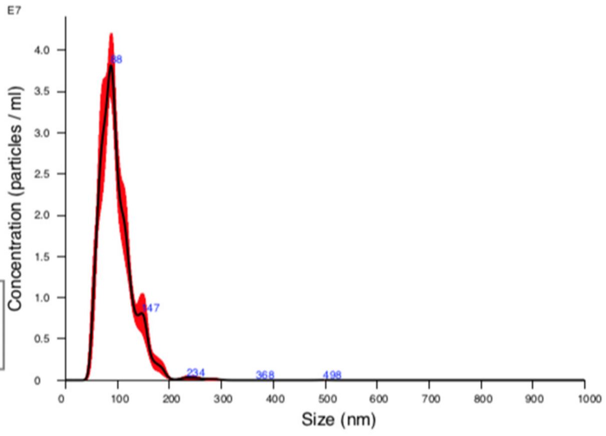

Averaged FTLA Concentration / Size for Experiment:
12SEC EVs 20x 25.11.20 2020-11-25 12-00-58
Error bars indicate + / -1 standard error of the mean

Included Files

12SEC EVs 20x 25.11.20 2020-11-25 12-01-21
12SEC EVs 20x 25.11.20 2020-11-25 12-02-09
12SEC EVs 20x 25.11.20 2020-11-25 12-02-50
12SEC EVs 20x 25.11.20 2020-11-25 12-03-37
12SEC EVs 20x 25.11.20 2020-11-25 12-04-36

Details

NTA Version:	NTA 3.4 Build 3.4.003
Script Used:	SOP Standard Measurement 12-00-58PM 25~
Time Captured:	12:00:58 25/11/2020
Operator:	Nadya24.11.20
Pre-treatment:	
Sample Name:	12SEC EVs 20x 25.11.20
Diluent:	PBS
Remarks:	

Capture Settings

Camera Type:	sCMOS
Laser Type:	Blue488
Camera Level:	13
Slider Shutter:	1232
Slider Gain:	175
FPS	25.0
Number of Frames:	749
Temperature:	24.2 - 24.3 ºC
Viscosity:	(Water) 0.902 - 0.905 cP
Dilution factor:	Dilution not recorded

Analysis Settings

Detect Threshold:	17
Blur Size:	Auto
Max Jump Distance:	Auto: 15.9 - 16.5 pix

Results

Stats: Merged Data

Mean:	97.7 nm
Mode:	87.3 nm
SD:	32.8 nm
D10:	63.6 nm
D50:	91.1 nm
D90:	141.6 nm

Stats: Mean +/- Standard Error

Mean:	97.8 +/- 1.4 nm
Mode:	85.7 +/- 4.0 nm
SD:	32.6 +/- 1.9 nm
D10:	63.4 +/- 1.7 nm
D50:	90.9 +/- 1.6 nm
D90:	141.0 +/- 3.4 nm
Concentration (Upgrade):	2.23e+09 +/- 7.61e+07 particles/ml
	66.5 +/- 2.6 particles/frame
	83.2 +/- 3.1 centres/frame

Concentration measurements may require some caution due to noise
See summary file for more info

Figure S13: PL, SEC fraction: 13

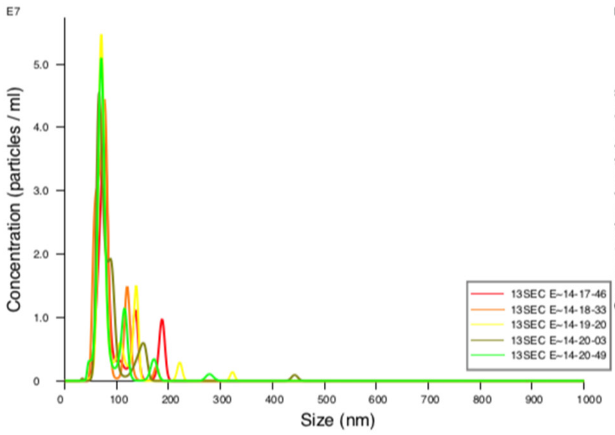

FTLA Concentration / Size graph for Experiment:
13SEC EVs 40x 25.11.20 2020-11-25 14-17-21

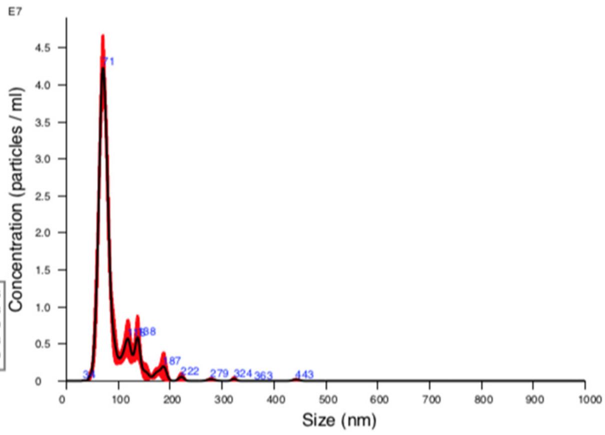

Averaged FTLA Concentration / Size for Experiment:
13SEC EVs 40x 25.11.20 2020-11-25 14-17-21
Error bars indicate + / -1 standard error of the mean

Included Files

13SEC EVs 40x 25.11.20 2020-11-25 14-17-46
13SEC EVs 40x 25.11.20 2020-11-25 14-18-33
13SEC EVs 40x 25.11.20 2020-11-25 14-19-20
13SEC EVs 40x 25.11.20 2020-11-25 14-20-03
13SEC EVs 40x 25.11.20 2020-11-25 14-20-49

Details

NTA Version:	NTA 3.4 Build 3.4.003
Script Used:	SOP Standard Measurement 02-17-21PM 25~
Time Captured:	14:17:21 25/11/2020
Operator:	Nadya24.11.20
Pre-treatment:	
Sample Name:	13SEC EVs 40x 25.11.20
Diluent:	PBS
Remarks:	

Capture Settings

Camera Type:	sCMOS
Laser Type:	Blue488
Camera Level:	14
Slider Shutter:	1259
Slider Gain:	245
FPS	25.0
Number of Frames:	749
Temperature:	24.4 - 24.6 ºC
Viscosity:	(Water) 0.897 - 0.900 cP
Dilution factor:	Dilution not recorded

Analysis Settings

Detect Threshold:	17
Blur Size:	Auto
Max Jump Distance:	Auto: 16.2 - 17.4 pix

Results

Stats: Merged Data

Mean:	88.5 nm
Mode:	70.6 nm
SD:	38.9 nm
D10:	61.7 nm
D50:	75.1 nm
D90:	135.7 nm

Stats: Mean +/- Standard Error

Mean:	88.7 +/- 3.0 nm
Mode:	71.8 +/- 1.9 nm
SD:	38.0 +/- 3.7 nm
D10:	62.1 +/- 1.3 nm
D50:	75.2 +/- 1.2 nm
D90:	139.5 +/- 11.1 nm
Concentration (Upgrade):	1.23e+09 +/- 3.86e+07 particles/ml
	34.8 +/- 2.1 particles/frame
	40.4 +/- 2.3 centres/frame

Figure S14: PL, SEC fraction: 14

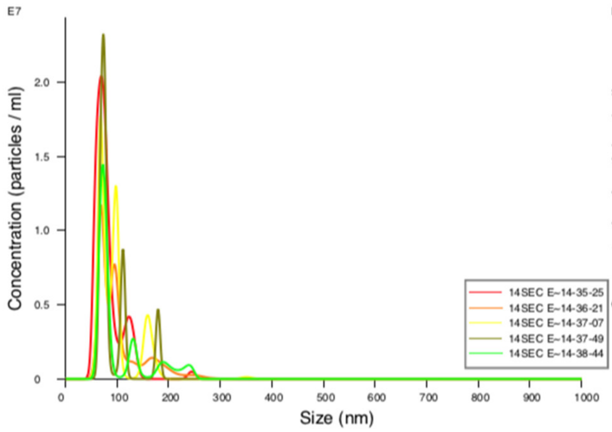

FTLA Concentration / Size graph for Experiment:
14SEC EVs 40x 25.11.20 2020-11-25 14-34-31

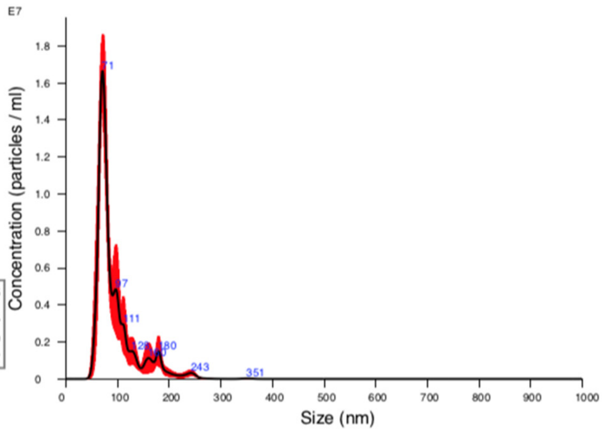

Averaged FTLA Concentration / Size for Experiment:
14SEC EVs 40x 25.11.20 2020-11-25 14-34-31
Error bars indicate + / -1 standard error of the mean

Included Files

14SEC EVs 40x 25.11.20 2020-11-25 14-35-25
14SEC EVs 40x 25.11.20 2020-11-25 14-36-21
14SEC EVs 40x 25.11.20 2020-11-25 14-37-07
14SEC EVs 40x 25.11.20 2020-11-25 14-37-49
14SEC EVs 40x 25.11.20 2020-11-25 14-38-44

Details

NTA Version:	NTA 3.4 Build 3.4.003
Script Used:	SOP Standard Measurement 02-34-31PM 25~
Time Captured:	14:34:31 25/11/2020
Operator:	Nadya24.11.20
Pre-treatment:	
Sample Name:	14SEC EVs 40x 25.11.20
Diluent:	PBS
Remarks:	

Capture Settings

Camera Type:	sCMOS
Laser Type:	Blue488
Camera Level:	14
Slider Shutter:	1259
Slider Gain:	245
FPS	25.0
Number of Frames:	749
Temperature:	24.8 ºC
Viscosity:	(Water) 0.891 - 0.893 cP
Dilution factor:	Dilution not recorded

Analysis Settings

Detect Threshold:	17
Blur Size:	Auto
Max Jump Distance:	Auto: 15.8 - 17.9 pix

Results

Stats: Merged Data	
Mean:	93.8 nm
Mode:	70.7 nm
SD:	39.9 nm
D10:	63.0 nm
D50:	78.3 nm
D90:	156.0 nm

Stats: Mean +/- Standard Error	
Mean:	95.3 +/- 4.1 nm
Mode:	70.5 +/- 1.0 nm
SD:	39.6 +/- 4.2 nm
D10:	63.8 +/- 1.6 nm
D50:	81.0 +/- 3.1 nm
D90:	164.7 +/- 12.7 nm
Concentration (Upgrade):	5.53e+08 +/- 6.03e+07 particles/ml
	17.0 +/- 1.1 particles/frame
	19.4 +/- 1.2 centres/frame

Figure S15: PL, SEC fraction: 15

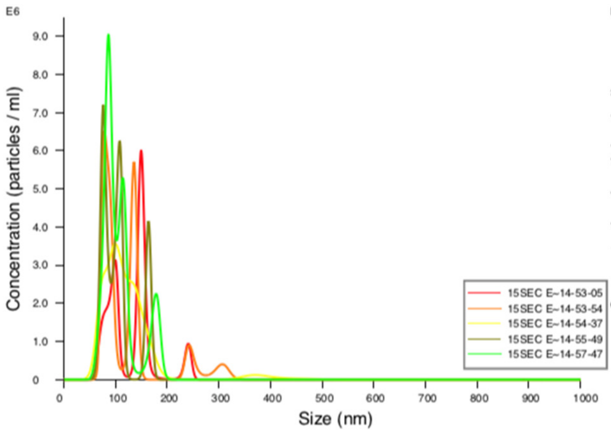

FTLA Concentration / Size graph for Experiment:
15SEC EVs 40x 25.11.20 2020-11-25 14-52-25

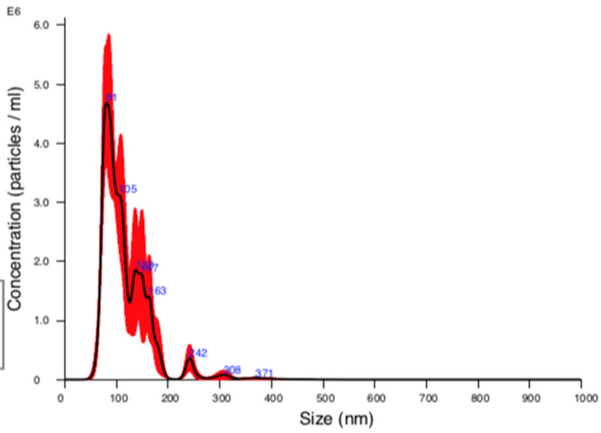

Averaged FTLA Concentration / Size for Experiment:
15SEC EVs 40x 25.11.20 2020-11-25 14-52-25
Error bars indicate + / -1 standard error of the mean

Included Files

15SEC EVs 40x 25.11.20 2020-11-25 14-53-05
15SEC EVs 40x 25.11.20 2020-11-25 14-53-54
15SEC EVs 40x 25.11.20 2020-11-25 14-54-37
15SEC EVs 40x 25.11.20 2020-11-25 14-55-49
15SEC EVs 40x 25.11.20 2020-11-25 14-57-47

Details

NTA Version:	NTA 3.4 Build 3.4.003
Script Used:	SOP Standard Measurement 02-52-25PM 25~
Time Captured:	14:52:25 25/11/2020
Operator:	Nadya24.11.20
Pre-treatment:	
Sample Name:	15SEC EVs 40x 25.11.20
Diluent:	PBS
Remarks:	

Capture Settings

Camera Type:	sCMOS
Laser Type:	Blue488
Camera Level:	13
Slider Shutter:	1232
Slider Gain:	175
FPS	25.0
Number of Frames:	749
Temperature:	24.4 - 24.7 °C
Viscosity:	(Water) 0.895 - 0.901 cP
Dilution factor:	Dilution not recorded

Analysis Settings

Detect Threshold:	17
Blur Size:	Auto
Max Jump Distance:	Auto: 14.4 - 16.1 pix

Results

Stats: Merged Data

Mean:	116.7 nm
Mode:	80.6 nm
SD:	48.4 nm
D10:	74.7 nm
D50:	103.6 nm
D90:	166.0 nm

Stats: Mean +/- Standard Error

Mean:	117.8 +/- 4.8 nm
Mode:	98.0 +/- 13.5 nm
SD:	46.1 +/- 6.4 nm
D10:	75.2 +/- 1.1 nm
D50:	109.4 +/- 8.4 nm
D90:	180.5 +/- 15.1 nm
Concentration (Upgrade):	2.90e+08 +/- 2.31e+07 particles/ml
	9.7 +/- 0.4 particles/frame
	10.6 +/- 0.4 centres/frame

Figure S16: PL, SEC fraction: 16-21

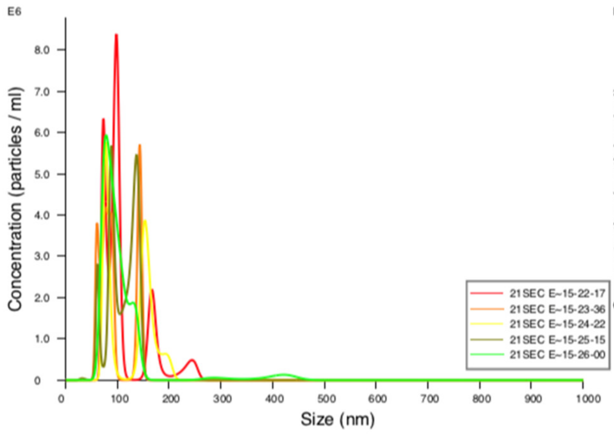

FTLA Concentration / Size graph for Experiment:
21SEC EVs 40x 25.11.20 2020-11-25 15-21-18

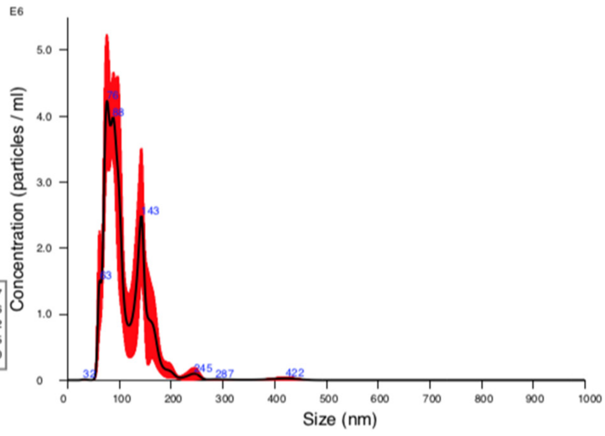

Averaged FTLA Concentration / Size for Experiment:
21SEC EVs 40x 25.11.20 2020-11-25 15-21-18
Error bars indicate + / -1 standard error of the mean

Included Files

21SEC EVs 40x 25.11.20 2020-11-25 15-22-17
21SEC EVs 40x 25.11.20 2020-11-25 15-23-36
21SEC EVs 40x 25.11.20 2020-11-25 15-24-22
21SEC EVs 40x 25.11.20 2020-11-25 15-25-15
21SEC EVs 40x 25.11.20 2020-11-25 15-26-00

Details

NTA Version:	NTA 3.4 Build 3.4.003
Script Used:	SOP Standard Measurement 03-21-18PM 25~
Time Captured:	15:21:18 25/11/2020
Operator:	Nadya24.11.20
Pre-treatment:	
Sample Name:	21SEC EVs 40x 25.11.20
Diluent:	PBS
Remarks:	

Capture Settings

Camera Type:	sCMOS
Laser Type:	Blue488
Camera Level:	13
Slider Shutter:	1232
Slider Gain:	175
FPS	25.0
Number of Frames:	749
Temperature:	23.7 - 23.9 °C
Viscosity:	(Water) 0.911 - 0.916 cP
Dilution factor:	Dilution not recorded

Analysis Settings

Detect Threshold:	17
Blur Size:	Auto
Max Jump Distance:	Auto: 13.8 - 17.5 pix

Results

Stats: Merged Data

Mean:	110.1 nm
Mode:	75.9 nm
SD:	46.0 nm
D10:	70.8 nm
D50:	95.7 nm
D90:	158.3 nm

Stats: Mean +/- Standard Error

Mean:	110.2 +/- 4.4 nm
Mode:	97.0 +/- 12.2 nm
SD:	42.1 +/- 6.5 nm
D10:	70.4 +/- 2.2 nm
D50:	106.0 +/- 10.5 nm
D90:	154.3 +/- 8.4 nm
Concentration (Upgrade):	2.42e+08 +/- 1.77e+07 particles/ml
	8.3 +/- 0.4 particles/frame
	8.8 +/- 0.4 centres/frame

Figure S17: PL-Dil, SEC fraction: 5-6

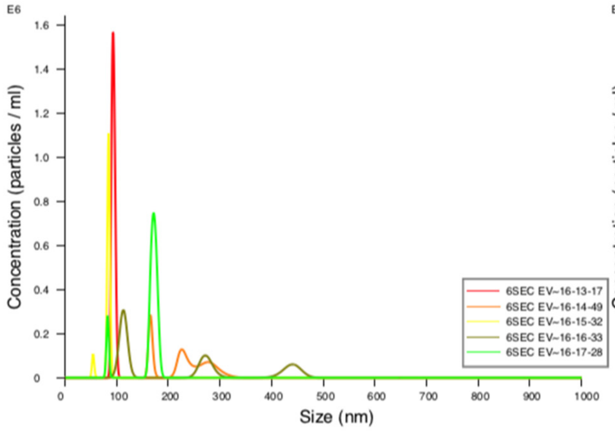

FTLA Concentration / Size graph for Experiment:
6SEC EVs 20x CM-DIL 50pM 25.11.20 2020-11-25 16-12-06

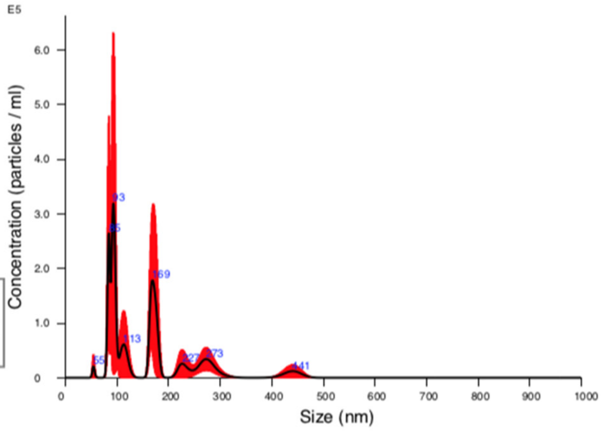

Averaged FTLA Concentration / Size for Experiment:
6SEC EVs 20x CM-DIL 50pM 25.11.20 2020-11-25 16-12-06
Error bars indicate + / -1 standard error of the mean

Included Files

6SEC EVs 20x CM-DIL 50pM 25.11.20 2020-11-25 16-13-17
6SEC EVs 20x CM-DIL 50pM 25.11.20 2020-11-25 16-14-49
6SEC EVs 20x CM-DIL 50pM 25.11.20 2020-11-25 16-15-32
6SEC EVs 20x CM-DIL 50pM 25.11.20 2020-11-25 16-16-33
6SEC EVs 20x CM-DIL 50pM 25.11.20 2020-11-25 16-17-28

Details

NTA Version:	NTA 3.4 Build 3.4.003
Script Used:	SOP Standard Measurement 04-12-06PM 25~
Time Captured:	16:12:06 25/11/2020
Operator:	Nadya24.11.20
Pre-treatment:	
Sample Name:	6SEC EVs 20x CM-DIL 50pM 25.11.20
Diluent:	PBS
Remarks:	

Capture Settings

Camera Type:	sCMOS
Laser Type:	Blue488
Camera Level:	15
Slider Shutter:	1206
Slider Gain:	245
FPS	25.0
Number of Frames:	749
Temperature:	25.1 ºC
Viscosity:	(Water) 0.885 - 0.886 cP
Dilution factor:	Dilution not recorded

Analysis Settings

Detect Threshold:	17
Blur Size:	Auto
Max Jump Distance:	Auto: 9.2 - 21.2 pix

Results

Stats: Merged Data

Mean:	160.1 nm
Mode:	92.9 nm
SD:	88.9 nm
D10:	84.7 nm
D50:	123.4 nm
D90:	277.0 nm

Stats: Mean +/- Standard Error

Mean:	158.8 +/- 31.5 nm
Mode:	125.4 +/- 18.2 nm
SD:	44.1 +/- 23.1 nm
D10:	104.7 +/- 15.5 nm
D50:	142.2 +/- 27.0 nm
D90:	219.4 +/- 66.3 nm
Concentration (Upgrade):	1.15e+07 +/- 1.23e+06 particles/ml
	0.7 +/- 0.1 particles/frame
	0.8 +/- 0.2 centres/frame

Concentration measurements may be unreliable
See summary file for more info

Figure S18: PL-Dil, SEC fraction: 7

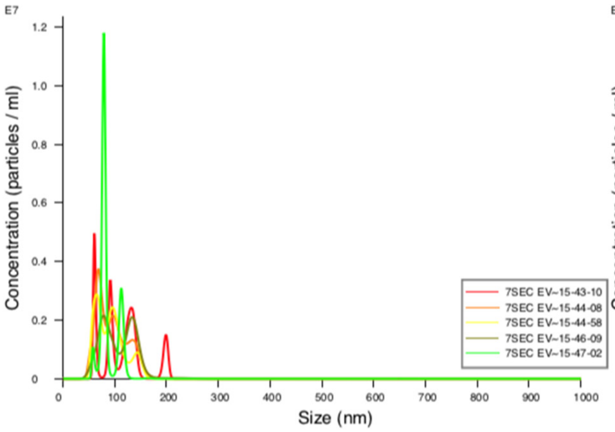

FTLA Concentration / Size graph for Experiment:
7SEC EVs 20x CM-DIL 50pM 25.11.20 2020-11-25 15-42-30

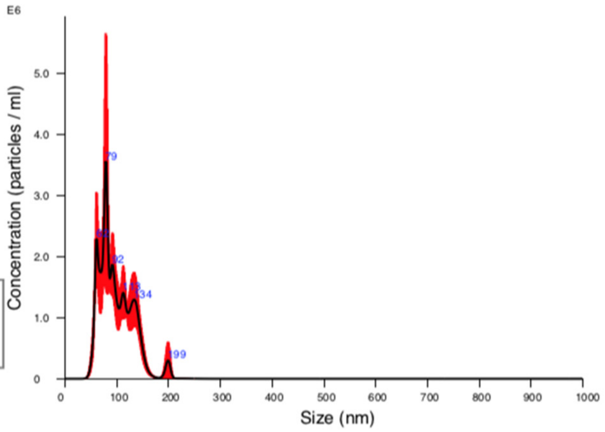

Averaged FTLA Concentration / Size for Experiment:
7SEC EVs 20x CM-DIL 50pM 25.11.20 2020-11-25 15-42-30
Error bars indicate + / -1 standard error of the mean

Included Files

7SEC EVs 20x CM-DIL 50pM 25.11.20 2020-11-25 15-43-10
7SEC EVs 20x CM-DIL 50pM 25.11.20 2020-11-25 15-44-08
7SEC EVs 20x CM-DIL 50pM 25.11.20 2020-11-25 15-44-58
7SEC EVs 20x CM-DIL 50pM 25.11.20 2020-11-25 15-46-09
7SEC EVs 20x CM-DIL 50pM 25.11.20 2020-11-25 15-47-02

Details

NTA Version:	NTA 3.4 Build 3.4.003
Script Used:	SOP Standard Measurement 03-42-30PM 25~
Time Captured:	15:42:30 25/11/2020
Operator:	Nadya24.11.20
Pre-treatment:	
Sample Name:	7SEC EVs 20x CM-DIL 50pM 25.11.20
Diluent:	PBS
Remarks:	

Capture Settings

Camera Type:	sCMOS
Laser Type:	Blue488
Camera Level:	15
Slider Shutter:	1206
Slider Gain:	245
FPS	25.0
Number of Frames:	749
Temperature:	24.4 - 24.6 ºC
Viscosity:	(Water) 0.897 - 0.900 cP
Dilution factor:	Dilution not recorded

Analysis Settings

Detect Threshold:	17
Blur Size:	Auto
Max Jump Distance:	Auto: 14.9 - 19.7 pix

Results

Stats: Merged Data

Mean:	98.2 nm
Mode:	78.8 nm
SD:	31.9 nm
D10:	62.1 nm
D50:	90.8 nm
D90:	139.9 nm

Stats: Mean +/- Standard Error

Mean:	98.6 +/- 4.9 nm
Mode:	70.3 +/- 3.6 nm
SD:	29.5 +/- 4.1 nm
D10:	64.6 +/- 2.6 nm
D50:	95.6 +/- 6.1 nm
D90:	145.6 +/- 13.2 nm
Concentration (Upgrade):	1.59e+08 +/- 7.71e+06 particles/ml
	8.2 +/- 0.5 particles/frame
	8.5 +/- 0.5 centres/frame

Figure S19: PL-Dil, SEC fraction: 8

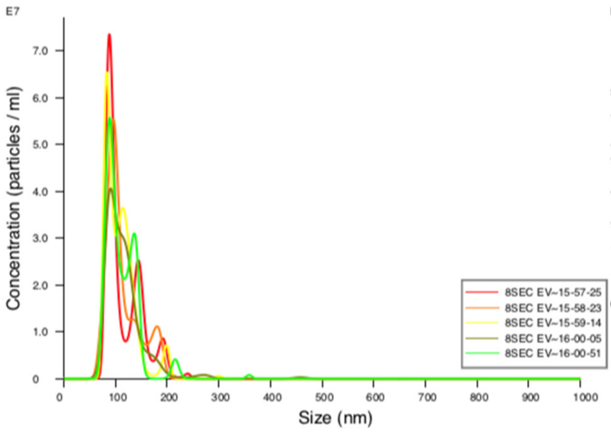

FTLA Concentration / Size graph for Experiment:
8SEC EVs 20x CM-DIL 50pM 25.11.20 2020-11-25 15-57-04

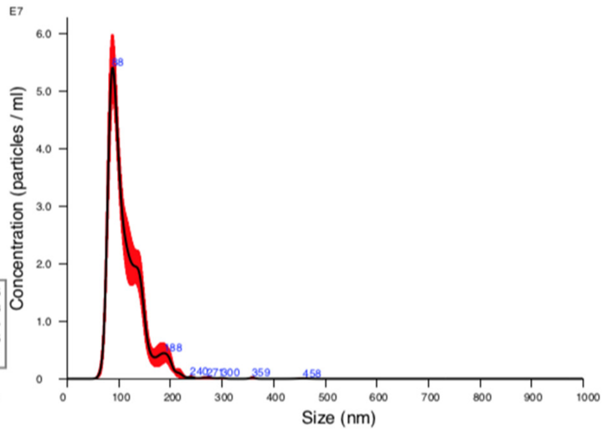

Averaged FTLA Concentration / Size for Experiment:
8SEC EVs 20x CM-DIL 50pM 25.11.20 2020-11-25 15-57-04
Error bars indicate + / -1 standard error of the mean

Included Files

8SEC EVs 20x CM-DIL 50pM 25.11.20 2020-11-25 15-57-25
8SEC EVs 20x CM-DIL 50pM 25.11.20 2020-11-25 15-58-23
8SEC EVs 20x CM-DIL 50pM 25.11.20 2020-11-25 15-59-14
8SEC EVs 20x CM-DIL 50pM 25.11.20 2020-11-25 16-00-05
8SEC EVs 20x CM-DIL 50pM 25.11.20 2020-11-25 16-00-51

Details

NTA Version:	NTA 3.4 Build 3.4.003
Script Used:	SOP Standard Measurement 03-57-04PM 25~
Time Captured:	15:57:04 25/11/2020
Operator:	Nadya24.11.20
Pre-treatment:	
Sample Name:	8SEC EVs 20x CM-DIL 50pM 25.11.20
Diluent:	PBS
Remarks:	

Capture Settings

Camera Type:	sCMOS
Laser Type:	Blue488
Camera Level:	13
Slider Shutter:	1232
Slider Gain:	175
FPS	25.0
Number of Frames:	749
Temperature:	25.1 ºC
Viscosity:	(Water) 0.885 - 0.886 cP
Dilution factor:	Dilution not recorded

Analysis Settings

Detect Threshold:	17
Blur Size:	Auto
Max Jump Distance:	Auto: 14.6 - 14.9 pix

Results

Stats: Merged Data

Mean:	112.4 nm
Mode:	87.9 nm
SD:	34.6 nm
D10:	80.6 nm
D50:	102.5 nm
D90:	153.3 nm

Stats: Mean +/- Standard Error

Mean:	112.5 +/- 1.3 nm
Mode:	89.1 +/- 2.0 nm
SD:	34.5 +/- 1.6 nm
D10:	80.6 +/- 0.6 nm
D50:	102.5 +/- 1.3 nm
D90:	154.7 +/- 5.8 nm
Concentration (Upgrade):	2.56e+09 +/- 5.73e+07 particles/ml
	119.5 +/- 2.7 particles/frame
	112.6 +/- 2.1 centres/frame

Figure S20: PL-Dil, SEC fraction: 9

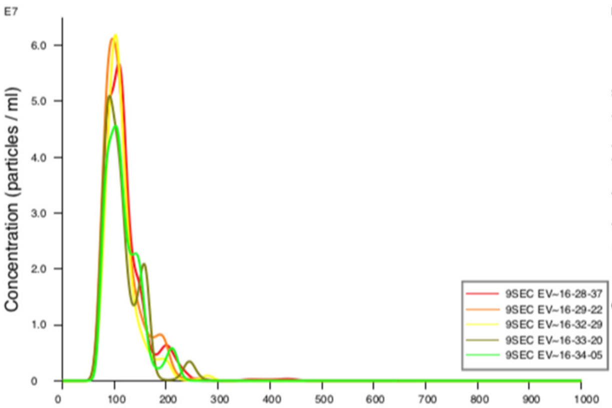

FTLA Concentration / Size graph for Experiment:
9SEC EVs 20x CM-DIL 50pM 25.11.20 2020-11-25 16-28-06

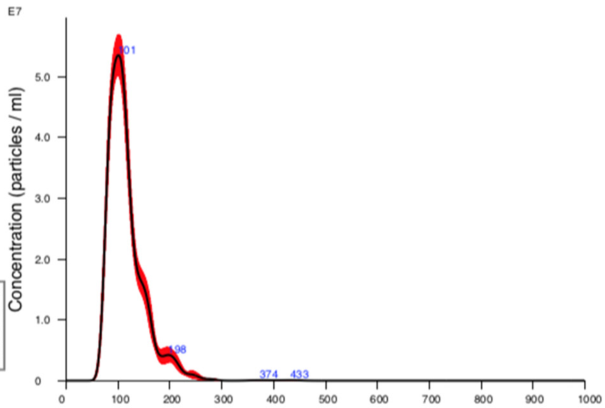

Averaged FTLA Concentration / Size for Experiment:
9SEC EVs 20x CM-DIL 50pM 25.11.20 2020-11-25 16-28-06
Error bars indicate + / -1 standard error of the mean

Included Files

9SEC EVs 20x CM-DIL 50pM 25.11.20 2020-11-25 16-28-37
9SEC EVs 20x CM-DIL 50pM 25.11.20 2020-11-25 16-29-22
9SEC EVs 20x CM-DIL 50pM 25.11.20 2020-11-25 16-32-29
9SEC EVs 20x CM-DIL 50pM 25.11.20 2020-11-25 16-33-20
9SEC EVs 20x CM-DIL 50pM 25.11.20 2020-11-25 16-34-05

Details

NTA Version:	NTA 3.4 Build 3.4.003
Script Used:	SOP Standard Measurement 04-28-06PM 25~
Time Captured:	16:28:06 25/11/2020
Operator:	Nadya24.11.20
Pre-treatment:	
Sample Name:	9SEC EVs 20x CM-DIL 50pM 25.11.20
Diluent:	PBS
Remarks:	

Capture Settings

Camera Type:	sCMOS
Laser Type:	Blue488
Camera Level:	13
Slider Shutter:	1232
Slider Gain:	175
FPS	25.0
Number of Frames:	749
Temperature:	25.4 ºC
Viscosity:	(Water) 0.879 - 0.880 cP
Dilution factor:	Dilution not recorded

Analysis Settings

Detect Threshold:	17
Blur Size:	Auto
Max Jump Distance:	Auto: 14.1 - 15.0 pix

Results

Stats: Merged Data

Mean:	116.2 nm
Mode:	100.7 nm
SD:	37.5 nm
D10:	80.5 nm
D50:	107.6 nm
D90:	161.5 nm

Stats: Mean +/- Standard Error

Mean:	116.1 +/- 1.1 nm
Mode:	100.1 +/- 3.1 nm
SD:	37.0 +/- 1.8 nm
D10:	80.6 +/- 0.8 nm
D50:	107.6 +/- 1.0 nm
D90:	161.4 +/- 2.8 nm
Concentration (Upgrade):	3.38e+09 +/- 1.54e+08 particles/ml
	175.5 +/- 10.7 particles/frame
	151.2 +/- 6.9 centres/frame

Figure S21: PL-Dil, SEC fraction: 10

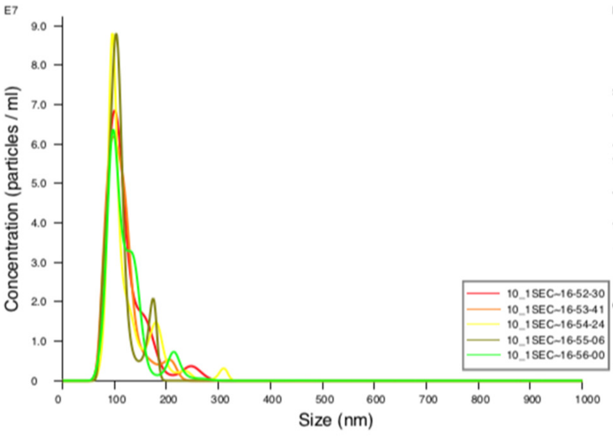

FTLA Concentration / Size graph for Experiment:
10_1SEC EVs 20x CM-DIL 50pM 25.11.20 2020-11-25 16-51-43

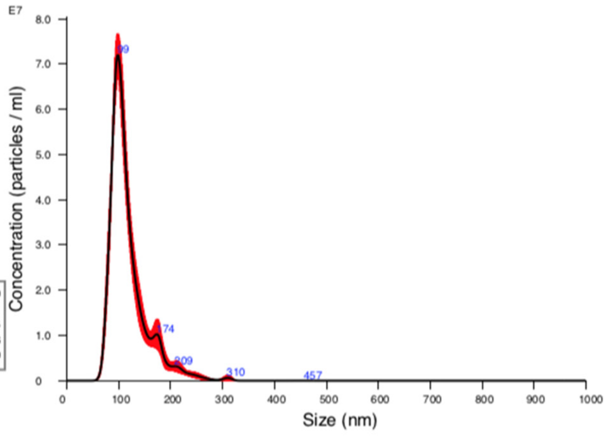

Averaged FTLA Concentration / Size for Experiment:
10_1SEC EVs 20x CM-DIL 50pM 25.11.20 2020-11-25 16-51-43
Error bars indicate + / -1 standard error of the mean

Included Files

10_1SEC EVs 20x CM-DIL 50pM 25.11.20 2020-11-25 16-52-30
10_1SEC EVs 20x CM-DIL 50pM 25.11.20 2020-11-25 16-53-41
10_1SEC EVs 20x CM-DIL 50pM 25.11.20 2020-11-25 16-54-24
10_1SEC EVs 20x CM-DIL 50pM 25.11.20 2020-11-25 16-55-06
10_1SEC EVs 20x CM-DIL 50pM 25.11.20 2020-11-25 16-56-00

Details

NTA Version:	NTA 3.4 Build 3.4.003
Script Used:	SOP Standard Measurement 04-51-43PM 25~
Time Captured:	16:51:43 25/11/2020
Operator:	Nadya24.11.20
Pre-treatment:	
Sample Name:	10_1SEC EVs 20x CM-DIL 50pM 25.11.20
Diluent:	PBS
Remarks:	

Capture Settings

Camera Type:	sCMOS
Laser Type:	Blue488
Camera Level:	12
Slider Shutter:	1200
Slider Gain:	125
FPS	25.0
Number of Frames:	749
Temperature:	25.6 - 25.7 ºC
Viscosity:	(Water) 0.876 - 0.877 cP
Dilution factor:	Dilution not recorded

Analysis Settings

Detect Threshold:	17
Blur Size:	Auto
Max Jump Distance:	Auto: 14.2 - 14.7 pix

Results

Stats: Merged Data

Mean:	117.8 nm
Mode:	98.3 nm
SD:	36.9 nm
D10:	84.5 nm
D50:	107.2 nm
D90:	168.9 nm

Stats: Mean +/- Standard Error

Mean:	117.8 +/- 2.4 nm
Mode:	99.0 +/- 1.3 nm
SD:	36.1 +/- 3.2 nm
D10:	84.5 +/- 0.9 nm
D50:	107.5 +/- 1.2 nm
D90:	165.9 +/- 5.5 nm
Concentration (Upgrade):	3.58e+09 +/- 1.00e+08 particles/ml
	140.6 +/- 6.5 particles/frame
	134.0 +/- 4.8 centres/frame

Figure S22: PL-Dil, SEC fraction: 11

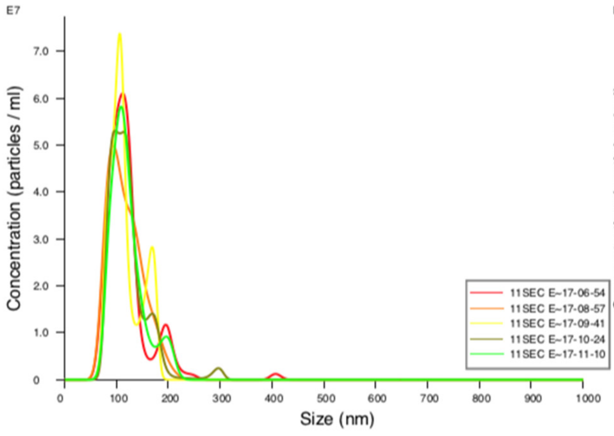

FTLA Concentration / Size graph for Experiment:
11SEC EVs 20x CM-DIL 50pM 25.11.20 2020-11-25 17-06-39

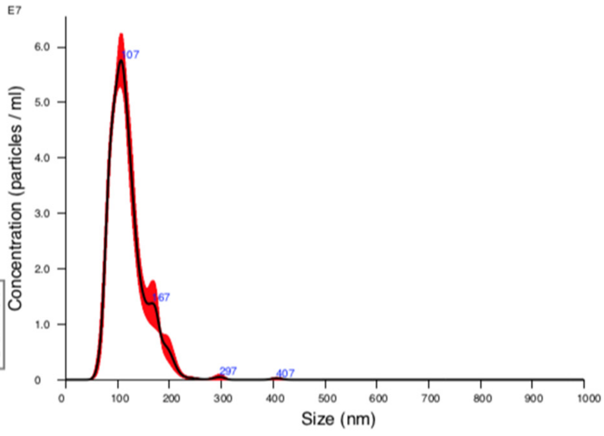

Averaged FTLA Concentration / Size for Experiment:
11SEC EVs 20x CM-DIL 50pM 25.11.20 2020-11-25 17-06-39
Error bars indicate + / -1 standard error of the mean

Included Files

11SEC EVs 20x CM-DIL 50pM 25.11.20 2020-11-25 17-06-54
11SEC EVs 20x CM-DIL 50pM 25.11.20 2020-11-25 17-08-57
11SEC EVs 20x CM-DIL 50pM 25.11.20 2020-11-25 17-09-41
11SEC EVs 20x CM-DIL 50pM 25.11.20 2020-11-25 17-10-24
11SEC EVs 20x CM-DIL 50pM 25.11.20 2020-11-25 17-11-10

Details

NTA Version:	NTA 3.4 Build 3.4.003
Script Used:	SOP Standard Measurement 05-06-39PM 25~
Time Captured:	17:06:39 25/11/2020
Operator:	Nadya24.11.20
Pre-treatment:	
Sample Name:	11SEC EVs 20x CM-DIL 50pM 25.11.20
Diluent:	PBS
Remarks:	

Capture Settings

Camera Type:	sCMOS
Laser Type:	Blue488
Camera Level:	12
Slider Shutter:	1200
Slider Gain:	125
FPS	25.0
Number of Frames:	749
Temperature:	25.1 - 25.2 ºC
Viscosity:	(Water) 0.884 - 0.887 cP
Dilution factor:	Dilution not recorded

Analysis Settings

Detect Threshold:	17
Blur Size:	Auto
Max Jump Distance:	Auto: 14.3 - 14.7 pix

Results

Stats: Merged Data

Mean:	119.2 nm
Mode:	106.5 nm
SD:	36.1 nm
D10:	82.5 nm
D50:	111.8 nm
D90:	168.4 nm

Stats: Mean +/- Standard Error

Mean:	119.2 +/- 0.9 nm
Mode:	103.7 +/- 3.5 nm
SD:	35.5 +/- 2.5 nm
D10:	82.7 +/- 1.5 nm
D50:	112.0 +/- 1.0 nm
D90:	170.7 +/- 3.1 nm
Concentration (Upgrade):	3.76e+09 +/- 8.95e+07 particles/ml
	148.3 +/- 4.6 particles/frame
	147.7 +/- 4.8 centres/frame

Figure S23: PL-Dil, SEC fraction: 12

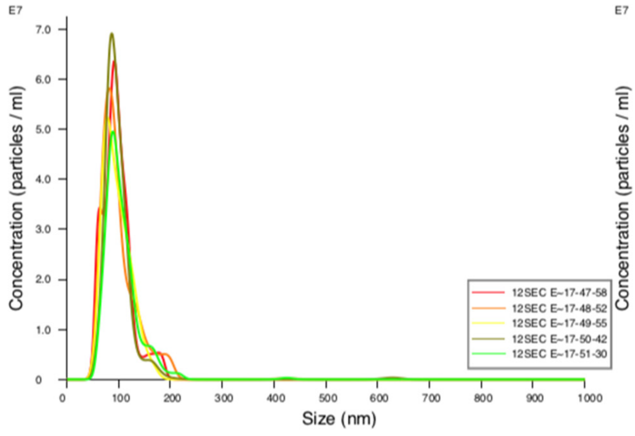

FTLA Concentration / Size graph for Experiment:
12SEC EVs 20x CM-DIL 50pM 25.11.20 2020-11-25 17-47-36

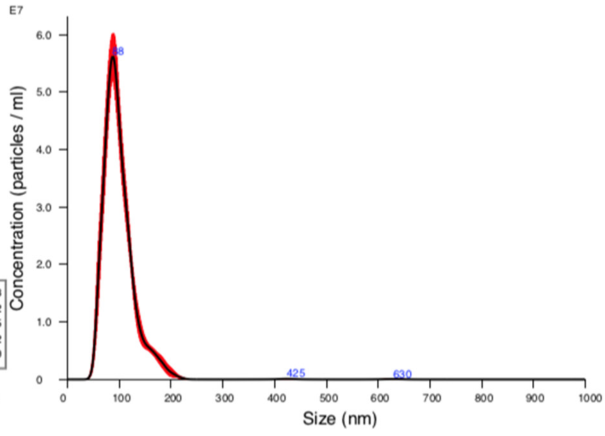

Averaged FTLA Concentration / Size for Experiment:
12SEC EVs 20x CM-DIL 50pM 25.11.20 2020-11-25 17-47-36
Error bars indicate + / -1 standard error of the mean

Included Files

12SEC EVs 20x CM-DIL 50pM 25.11.20 2020-11-25 17-47-58
12SEC EVs 20x CM-DIL 50pM 25.11.20 2020-11-25 17-48-52
12SEC EVs 20x CM-DIL 50pM 25.11.20 2020-11-25 17-49-55
12SEC EVs 20x CM-DIL 50pM 25.11.20 2020-11-25 17-50-42
12SEC EVs 20x CM-DIL 50pM 25.11.20 2020-11-25 17-51-30

Details

NTA Version:	NTA 3.4 Build 3.4.003
Script Used:	SOP Standard Measurement 05-47-36PM 25~
Time Captured:	17:47:36 25/11/2020
Operator:	Nadya24.11.20
Pre-treatment:	
Sample Name:	12SEC EVs 20x CM-DIL 50pM 25.11.20
Diluent:	PBS
Remarks:	

Capture Settings

Camera Type:	sCMOS
Laser Type:	Blue488
Camera Level:	13
Slider Shutter:	1232
Slider Gain:	175
FPS	25.0
Number of Frames:	749
Temperature:	25.2 - 25.3 ºC
Viscosity:	(Water) 0.882 - 0.884 cP
Dilution factor:	Dilution not recorded

Analysis Settings

Detect Threshold:	17
Blur Size:	Auto
Max Jump Distance:	Auto: 15.3 - 16.1 pix

Results

Stats: Merged Data

Mean:	99.8 nm
Mode:	87.6 nm
SD:	35.7 nm
D10:	67.6 nm
D50:	93.5 nm
D90:	136.9 nm

Stats: Mean +/- Standard Error

Mean:	100.0 +/- 1.6 nm
Mode:	85.6 +/- 2.4 nm
SD:	35.0 +/- 3.4 nm
D10:	68.0 +/- 1.8 nm
D50:	93.6 +/- 1.2 nm
D90:	137.0 +/- 5.0 nm
Concentration (Upgrade):	3.15e+09 +/- 1.08e+08 particles/ml
	114.9 +/- 5.8 particles/frame
	122.7 +/- 6.0 centres/frame

Figure S24: PL-Dil, SEC fraction: 13

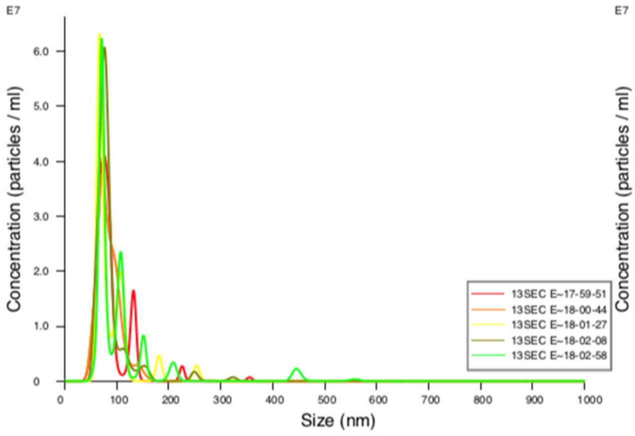

FTLA Concentration / Size graph for Experiment:
13SEC EVs 40x CM-DIL 50pM 25.11.20 2020-11-25 17-59-29

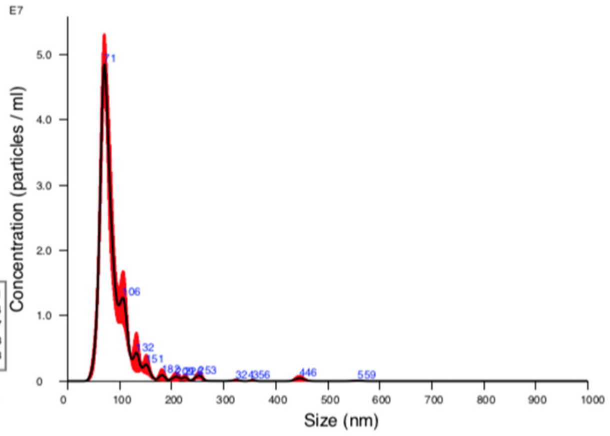

Averaged FTLA Concentration / Size for Experiment:
13SEC EVs 40x CM-DIL 50pM 25.11.20 2020-11-25 17-59-29
Error bars indicate + / -1 standard error of the mean

Included Files

13SEC EVs 40x CM-DIL 50pM 25.11.20 2020-11-25 17-59-51
13SEC EVs 40x CM-DIL 50pM 25.11.20 2020-11-25 18-00-44
13SEC EVs 40x CM-DIL 50pM 25.11.20 2020-11-25 18-01-27
13SEC EVs 40x CM-DIL 50pM 25.11.20 2020-11-25 18-02-08
13SEC EVs 40x CM-DIL 50pM 25.11.20 2020-11-25 18-02-58

Details

NTA Version:	NTA 3.4 Build 3.4.003
Script Used:	SOP Standard Measurement 05-59-29PM 25~
Time Captured:	17:59:29 25/11/2020
Operator:	Nadya24.11.20
Pre-treatment:	
Sample Name:	13SEC EVs 40x CM-DIL 50pM 25.11.20
Diluent:	PBS
Remarks:	

Capture Settings

Camera Type:	sCMOS
Laser Type:	Blue488
Camera Level:	14
Slider Shutter:	1259
Slider Gain:	245
FPS	25.0
Number of Frames:	749
Temperature:	25.2 - 25.3 ºC
Viscosity:	(Water) 0.883 - 0.885 cP
Dilution factor:	Dilution not recorded

Analysis Settings

Detect Threshold:	17
Blur Size:	Auto
Max Jump Distance:	Auto: 16.5 - 17.5 pix

Results

Stats: Merged Data

Mean:	90.5 nm
Mode:	70.6 nm
SD:	46.6 nm
D10:	61.9 nm
D50:	77.6 nm
D90:	126.0 nm

Stats: Mean +/- Standard Error

Mean:	90.8 +/- 4.1 nm
Mode:	72.2 +/- 1.6 nm
SD:	42.7 +/- 9.1 nm
D10:	61.7 +/- 1.1 nm
D50:	77.5 +/- 1.1 nm
D90:	125.7 +/- 7.9 nm
Concentration (Upgrade):	1.65e+09 +/- 5.05e+07 particles/ml
	51.7 +/- 0.7 particles/frame
	60.3 +/- 1.0 centres/frame

Figure S25: PL-Dil, SEC fraction: 14

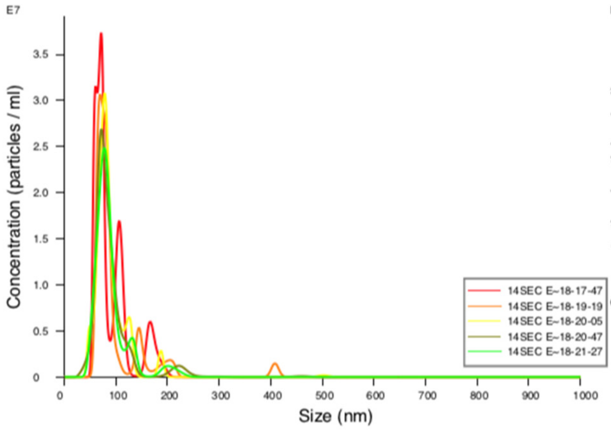

FTLA Concentration / Size graph for Experiment:
14SEC EVs 40x CM-DIL 50pM 25.11.20 2020-11-25 18-17-20

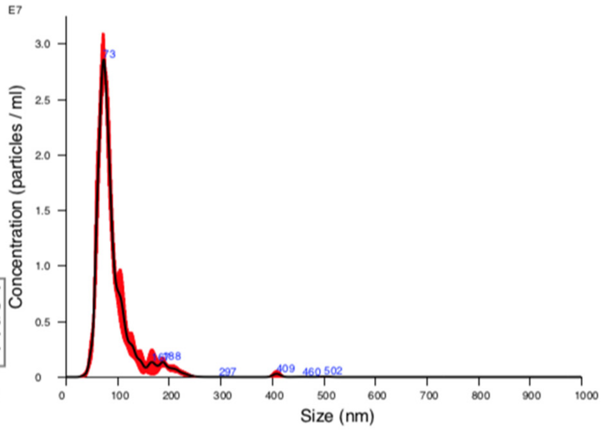

Averaged FTLA Concentration / Size for Experiment:
14SEC EVs 40x CM-DIL 50pM 25.11.20 2020-11-25 18-17-20
Error bars indicate + / -1 standard error of the mean

Included Files

14SEC EVs 40x CM-DIL 50pM 25.11.20 2020-11-25 18-17-47
14SEC EVs 40x CM-DIL 50pM 25.11.20 2020-11-25 18-19-19
14SEC EVs 40x CM-DIL 50pM 25.11.20 2020-11-25 18-20-05
14SEC EVs 40x CM-DIL 50pM 25.11.20 2020-11-25 18-20-47
14SEC EVs 40x CM-DIL 50pM 25.11.20 2020-11-25 18-21-27

Details

NTA Version:	NTA 3.4 Build 3.4.003
Script Used:	SOP Standard Measurement 06-17-20PM 25~
Time Captured:	18:17:20 25/11/2020
Operator:	Nadya24.11.20
Pre-treatment:	
Sample Name:	14SEC EVs 40x CM-DIL 50pM 25.11.20
Diluent:	PBS
Remarks:	

Capture Settings

Camera Type:	sCMOS
Laser Type:	Blue488
Camera Level:	14
Slider Shutter:	1259
Slider Gain:	245
FPS	25.0
Number of Frames:	749
Temperature:	25.0 - 25.1 ºC
Viscosity:	(Water) 0.887 - 0.889 cP
Dilution factor:	Dilution not recorded

Analysis Settings

Detect Threshold:	17
Blur Size:	Auto
Max Jump Distance:	Auto: 16.0 - 17.6 pix

Results

Stats: Merged Data

Mean:	91.0 nm
Mode:	72.7 nm
SD:	43.4 nm
D10:	60.6 nm
D50:	78.8 nm
D90:	132.4 nm

Stats: Mean +/- Standard Error

Mean:	91.0 +/- 1.9 nm
Mode:	73.8 +/- 1.8 nm
SD:	42.4 +/- 4.9 nm
D10:	60.7 +/- 0.8 nm
D50:	78.7 +/- 1.0 nm
D90:	137.6 +/- 8.2 nm
Concentration (Upgrade):	1.14e+09 +/- 4.77e+07 particles/ml
	37.8 +/- 2.4 particles/frame
	44.4 +/- 2.6 centres/frame

Concentration measurements may require some caution due to noise
See summary file for more info

Figure S26: PL-DiI, SEC fraction: 15

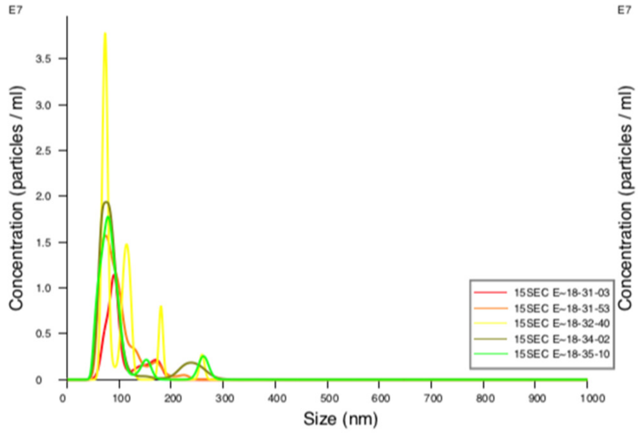

FTLA Concentration / Size graph for Experiment:
15SEC EVs 40x CM-DIL 50pM 25.11.20 2020-11-25 18-30-29

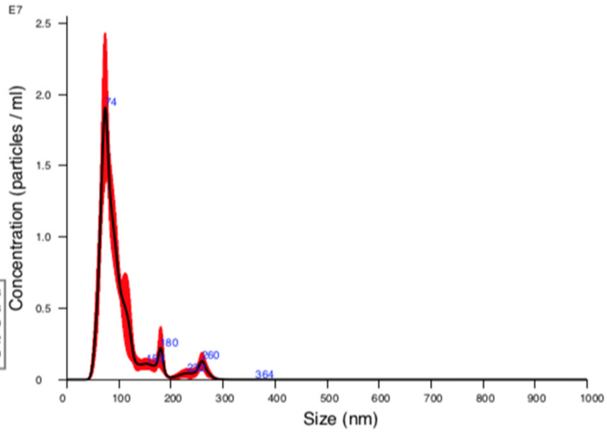

Averaged FTLA Concentration / Size for Experiment:
15SEC EVs 40x CM-DIL 50pM 25.11.20 2020-11-25 18-30-29
Error bars indicate + / -1 standard error of the mean

Included Files

15SEC EVs 40x CM-DIL 50pM 25.11.20 2020-11-25 18-31-03
15SEC EVs 40x CM-DIL 50pM 25.11.20 2020-11-25 18-31-53
15SEC EVs 40x CM-DIL 50pM 25.11.20 2020-11-25 18-32-40
15SEC EVs 40x CM-DIL 50pM 25.11.20 2020-11-25 18-34-02
15SEC EVs 40x CM-DIL 50pM 25.11.20 2020-11-25 18-35-10

Details

NTA Version:	NTA 3.4 Build 3.4.003
Script Used:	SOP Standard Measurement 06-30-29PM 25~
Time Captured:	18:30:29 25/11/2020
Operator:	Nadya24.11.20
Pre-treatment:	
Sample Name:	15SEC EVs 40x CM-DIL 50pM 25.11.20
Diluent:	PBS
Remarks:	

Capture Settings

Camera Type:	sCMOS
Laser Type:	Blue488
Camera Level:	14
Slider Shutter:	1259
Slider Gain:	245
FPS	25.0
Number of Frames:	749
Temperature:	25.0 - 25.1 ºC
Viscosity:	(Water) 0.886 - 0.889 cP
Dilution factor:	Dilution not recorded

Analysis Settings

Detect Threshold:	17
Blur Size:	Auto
Max Jump Distance:	Auto: 15.7 - 16.8 pix

Results

Stats: Merged Data

Mean:	99.9 nm
Mode:	73.6 nm
SD:	46.5 nm
D10:	64.2 nm
D50:	84.3 nm
D90:	165.1 nm

Stats: Mean +/- Standard Error

Mean:	100.2 +/- 1.3 nm
Mode:	78.2 +/- 3.2 nm
SD:	44.4 +/- 4.7 nm
D10:	65.0 +/- 2.2 nm
D50:	84.7 +/- 2.3 nm
D90:	172.8 +/- 13.1 nm
Concentration (Upgrade):	8.02e+08 +/- 9.02e+07 particles/ml
	28.7 +/- 3.1 particles/frame
	31.8 +/- 3.5 centres/frame

Figure S27: PL-Dil, SEC fraction: 16-21

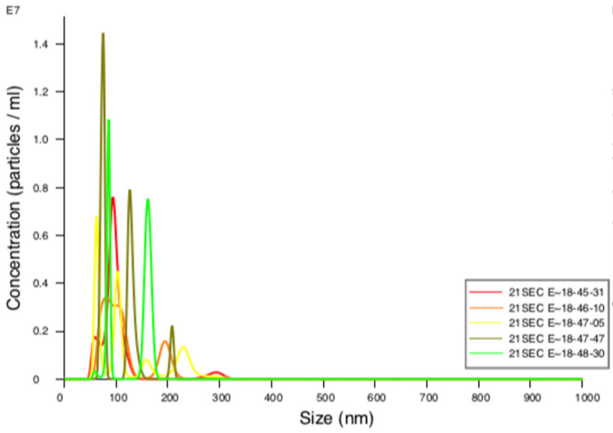

FTLA Concentration / Size graph for Experiment:
21SEC EVs 40x CM-DIL 50pM 25.11.20 2020 2020-11-25 18-45--17

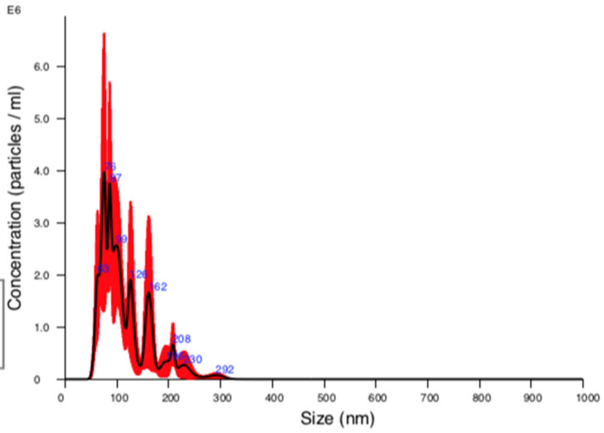

Averaged FTLA Concentration / Size for Experiment:
21SEC EVs 40x CM-DIL 50pM 25.11.20 2020 2020-11-25 18-45--17
Error bars indicate + / -1 standard error of the mean

Included Files

21SEC EVs 40x CM-DIL 50pM 25.11.20 2020 2020-11-25 18-45-31
21SEC EVs 40x CM-DIL 50pM 25.11.20 2020 2020-11-25 18-46-10
21SEC EVs 40x CM-DIL 50pM 25.11.20 2020 2020-11-25 18-47-05
21SEC EVs 40x CM-DIL 50pM 25.11.20 2020 2020-11-25 18-47-47
21SEC EVs 40x CM-DIL 50pM 25.11.20 2020 2020-11-25 18-48-30

Details

NTA Version:	NTA 3.4 Build 3.4.003
Script Used:	SOP Standard Measurement 06-45-17PM 25~
Time Captured:	18:45:17 25/11/2020
Operator:	Nadya24.11.20
Pre-treatment:	
Sample Name:	21SEC EVs 40x CM-DIL 50pM 25.11.20 2020
Diluent:	PBS
Remarks:	

Capture Settings

Camera Type:	sCMOS
Laser Type:	Blue488
Camera Level:	13
Slider Shutter:	1232
Slider Gain:	175
FPS	25.0
Number of Frames:	749
Temperature:	24.6 - 24.8 °C
Viscosity:	(Water) 0.893 - 0.896 cP
Dilution factor:	Dilution not recorded

Analysis Settings

Detect Threshold:	17
Blur Size:	Auto
Max Jump Distance:	Auto: 14.1 - 23.7 pix

Results

Stats: Merged Data

Mean:	114.3 nm
Mode:	75.1 nm
SD:	48.7 nm
D10:	67.9 nm
D50:	98.8 nm
D90:	185.3 nm

Stats: Mean +/- Standard Error

Mean:	115.1 +/- 5.6 nm
Mode:	79.5 +/- 5.3 nm
SD:	46.6 +/- 5.4 nm
D10:	70.1 +/- 3.7 nm
D50:	106.5 +/- 12.7 nm
D90:	170.8 +/- 20.5 nm
Concentration (Upgrade):	2.34e+08 +/- 1.14e+07 particles/ml
	8.7 +/- 0.1 particles/frame
	9.5 +/- 0.2 centres/frame
